# Isolation, characterization, therapeutic potency, and genomic analysis of a novel bacteriophage vB_KshKPC-M against carbapenemase-producing *Klebsiella pneumoniae* strains (CRKP) isolated from Ventilator-associated pneumoniae (VAP) infection of COVID-19 patients

**DOI:** 10.1186/s12941-023-00567-1

**Published:** 2023-02-24

**Authors:** Mehrdad Mohammadi, Mahmood Saffari, Seyed Davar Siadat, Seyed Hossein Hejazi, Mohammad Shayestehpour, Mitra Motallebi, Milad Eidi

**Affiliations:** 1 Department of Microbiology and Immunology, Faculty of Medicine, Kashan University of Medical Sciences, Pezeshk Blvd, Qotbe Ravandi Blvd, Kashan, 8715973449 Iran; 2 Tuberculosis and Pulmonary Research Department, Pasteur Institute of Iran, Tehran, Iran; 3 Microbiology Research Center (MRC), Pasteur Institute of Iran, Tehran, Iran; 4 Department of Parasitology and Mycology, School of Medicine, Isfahan University of Medical Sciences, Isfahan, Iran; 5 Department of Medical Genetics, Faculty of Medical Sciences, Tarbiat Modares University, Tehran, Iran

**Keywords:** *Klebsiella pneumoniae*, Carbapenemase, Bacteriophage, Phage Therapy, Whole genome sequencing, Antibiotic resistance, Infection

## Abstract

**Background:**

Carbapenem-resistant *Klebsiella pneumoniae* (CRKP) is a significant clinical problem, given the lack of therapeutic options. The CRKP strains have emerged as an essential worldwide healthcare issue during the last 10 years. Global expansion of the CRKP has made it a significant public health hazard. We must consider to novel therapeutic techniques. Bacteriophages are potent restorative cases against infections with multiple drug-resistant bacteria. The Phages offer promising prospects for the treatment of CRKP infections.

**Objective:**

In this study, a novel *K. pneumoniae* phage vB_KshKPC-M was isolated, characterized, and sequenced, which was able to infect and lyse Carbapenem-resistant *K. pneumoniae* host specifically.

**Methods:**

One hundred clinical isolates of *K. pneumoniae* were collected from patients with COVID-19 associated with ventilator-associated acute pneumonia hospitalized at Shahid Beheshti Hospital, Kashan, Iran, from 2020 to 2021. Initially, all samples were cultured, and bacterial isolates identified by conventional biochemical tests, and then the *ureD* gene was used by PCR to confirm the isolates. The Antibiotic susceptibility test in the disc diffusion method and Minimum inhibitory concentrations for Colistin was done and interpreted according to guidelines. Phenotypic and molecular methods determined the Carbapenem resistance of isolates. The *blaKPC*, *blaNDM*, and *blaOXA-23* genes were amplified for this detection. Biofilm determination of CRKP isolates was performed using a quantitative microtiter plate (MTP) method. The phage was isolated from wastewater during the summer season at a specific position from Beheshti Hospital (Kashan, Iran). The sample was processed and purified against the bacterial host, a CRKP strain isolated from a patient suffering from COVID-19 pneumoniae and resistance to Colistin with high potency for biofilm production. This isolate is called Kp100. The separated phages were diluted and titration by the double overlay agar plaque assay. The separate Phage is concentrated with 10% PEG and stored at −80 °C until use. The phage host range was identified by the spot test method. The purified phage morphology was determined using a transmission electron microscope. The phage stability tests (pH and temperature) were analyzed. The effect of cationic ions on phage adsorption was evaluated. The optimal titer of bacteriophage was determined to reduce the concentration of the CRKP strain. One-step growth assays were performed to identify the purified phage burst’s latent cycle and size. The SDS-PAGE was used for phage proteins analysis. Phage DNA was extracted by chloroform technique, and the whole genome of lytic phage was sequenced using Illumina HiSeq technology (Illumina, San Diego, CA). For quality assurance and preprocessing, such as trimming, Geneious Prime 2021.2.2 and Spades 3.9.0. The whole genome sequence of the lytic phage is linked to the GenBank database accession number. RASTtk—v1.073 was used to predict and annotate the ORFs. Prediction of ORF was performed using PHASTER software. ResFinder is used to assess the presence of antimicrobial resistance and virulence genes in the genome. The tRNAs can-SE v2.0.6 is used to determine the presence of tRNA in the genome. Linear genome comparisons of phages and visualization of coding regions were performed using Easyfig 2.2.3 and Mauve 2.4.0. Phage lifestyles were predicted using the program PHACTS. Phylogenetic analysis and amino acid sequences of phage core proteins, such as the major capsid protein. Phylogenies were reconstructed using the Neighbor-Joining method with 1000 bootstrap repeat. HHpred software was used to predict depolymerase. In this study, GraphPad Prism version 9.1 was used for the statistical analysis. Student’s t-test was used to compare the sets and the control sets, and the significance level was set at P ≤ 0.05.

**Results:**

Phage vB_KshKPC-M is assigned to the *Siphoviridae*, order *Caudovirales*. It was identified as a linear double-stranded DNA phage of 54,378 bp with 50.08% G + C content, had a relatively broad host range (97.7%), a short latency of 20 min, and a high burst size of 260 PFU/cell, and was maintained stable at different pH (3–11) and temperature (45–65 °C). The vB_KshKPC-M genome contains 91 open-reading frames. No tRNA, antibiotic resistance, toxin, virulence-related genes, or lysogen-forming gene clusters were detected in the phage genome.

Comparative genomic analysis revealed that phage vB_KshKPC-M has sequence similarity to the *Klebsiella* phages, phage 13 (NC_049844.1), phage Sushi (NC_028774.1), phage vB_KpnD_PeteCarol (OL539448.1) and phage PWKp14 (MZ634345.1).

**Conclusion:**

The broad host range and antibacterial activity make it a promising candidate for future phage therapy applications. The isolated phage was able to lyse most of the antibiotic-resistant clinical isolates. Therefore, this phage can be used alone or as a phage mixture in future studies to control and inhibit respiratory infections caused by these bacteria, especially in treating respiratory infections caused by resistant strains in sick patients.

## Introduction

*Klebsiella pneumoniae* is an opportunistic pathogen. It can colonize the skin and mucous membranes of humans and cause severe nosocomial infections, including pneumonia, meningitis, liver abscesses, urinary tract infections, and wound infections, primarily in immunocompromised individuals [1, 2]. The control of *K. pneumoniae* nosocomial diseases has become more complicated due to increasing antibiotic resistance, prolonged hospital stays, high mortality rates, and the high cost of treating patients [1, 3]. The two main types of antibiotic resistance mechanisms in *K. pneumoniae* include extended-spectrum lactamase (ESBL) production and carbapenem-resistant *K. pneumoniae* (CRKP) [4].

CRKP is a significant challenge due to its antibiotic resistance and increasing prevalence [5]. Nowadays, the global problem of antibiotic resistance is becoming more acute. CRKP strains are resistant to all carbapenem antibiotics [6]. Examples of carbapenems are imipenem, meropenem, doripenem, ertapenem, panipenem, and biapenem [7]. The antibiotics of the last series are tigecycline and Colistin [8]. However, CRKP strains have also shown resistance to Colistin and tigecycline [8].

*K. pneumoniae*, particularly carbapenem-resistance *K. pneumonia* (CRKP) infection, is the primary factor of mortality among the aged in the ICU, and its’ effect throughout the COVID-19 pandemic [9]. Because of commencing the pandemic, various authors have determined the relationship between COVID-19 and carbapenemase-producing *Klebsiella pneumoniae*, emphasizing that these infections may have the ability to confuse the term COVID-19 [10] critically. In the ICU, the prevalence of CRKP infections has increased meaningfully in the COVID-19 period compared to the non-COVID time (3.8%) [11]. The spread of multidrug-resistant (MDR) *Klebsiella *during the COVID-19 period was expedited by the intensified overuse of antibiotics [12]. International reports determined that nearly 70% of hospitalized patients with COVID-19 get antibiotics, despite a lack of evidence of bacterial coinfections [11]. With severe COVID-19 cases, the percentage of CRKP infections increased, leading to a higher mortality rate (30–70%) [9]. Conversely, the coinfections can contribute to a poor prognosis for patients with COVID-19, especially for high-risk populations such as elderly patients. Immunosuppressive treatments can lead to the unfavorable evolution of patients coinfected with COVID-19 and CRKP infections [9, 11, 12].

Alternative therapies have been introduced to combat this antibiotic-resistant pathogen. A new alternative method for treating carbapenem-resistant *K. pneumoniae* strains is bacteriophage therapy. Bacteriophage therapy has been proposed as a new strategy to prevent infections caused by *K. pneumoniae*, such as respiratory infections, liver abscesses, and bacteremia [13, 14].

Unfortunately, phage therapy was forgotten after the revolution in antibiotic discovery, a phenomenon due to technical limitations. However, the rate of development of bacterial drug resistance is much faster than the development of antibiotics, so phage therapy techniques are again attracting worldwide attention [15, 16]. Compared to antibiotics, phages are very specific and reduce secondary infections. Current research has shown that bacteriophages tremendously impact treating antibiotic-resistant bacterial infections. Bacteriophages are strain specific and may be a good choice for alternative therapeutic systems [17–19]. Advantages of phage therapy include (i) their strain-specificity, which means they can infect only a single species of their bacterial host, (ii) their low natural toxicity, as because they eliminate the bacterial host without disrupting normal human cells and flora, (iii) the unlikelihood of cross-resistance to antibiotics, (iv) their anti-biofilm activity, and (v) the immense natural reserve of different bacteriophages, which offers numerous treatment options and potential combination cocktails [20–23].

This study aimed to isolate, characterize, and purify whole genome sequencing and anti-biofilm effects against CRKP isolate from patients with COVID-19 and ventilator-associated acute pneumonia.

## Materials and methods

### Bacterial isolation, biochemical and molecular identification

The 103 clinical isolates were collected from patients with COVID-19 associated with ventilator-associated acute pneumonia. All samples contained tracheal secretions. These respiratory samples collected from COVID-19 patients hospitalized at Shahid Beheshti Hospital, Kashan, Iran, from 2020 to 2021.

The Ethics Committee of Kashan University of Medical Sciences, Kashan, Iran, approved this study (code: IR.KAUMS.MEDNT.REC.1400.023). Sampling and data collection were performed under the supervision of this committee. All participants provided written informed consent.

Initially, all samples were cultured on more usable mediums, including MacConkey agar and blood agar, and incubated overnight at 37 °C. The all bacterial isolates identified with conventional biochemical reactions. For molecular confirmation of isolates by PCR, the *ureD* gene was used. The DNA of the bacterial isolates was extracted by boiling. Then, to detect the *ureD* gene, we used the forward and reverse primer of Ghanizadeh et al. to amplify 243 base pairs (bp) of the *ureD* gene [24].

### Antibiotic susceptibility test

First, 3–5 individual colonies were selected from blood agar and solvent in sterilized normal saline to prepare a 0.5 McFarland standard (density of a bacterial suspension 1.5 × 10^8^ colonies forming unit). Then the bacterial cells cultured on Muller-Hinton agar (MHA). The antibiotics used to determine susceptibility, including trimethoprim/sulfamethoxazole (1.25/23.75 μg), amoxicillin-clavulanate (20/10 μg), piperacillin/tazobactam (100/10 μg), cefepime (30 μg), cefotaxime (30 μg), ciprofloxacin (5 μg), Imipenem (10 μg), Ertapenem (10 μg) and Meropenem (10 μg), Tobramycin (10 μg), Gentamicin (10 μg) and Aztreonam (30 μg). The diameter of the inhibition zone was measured after incubation for 16–18 h at 35 ± two °C, and interpreted according to the CLSI (2021) M100-S31 guidelines [25]. Minimum inhibitory concentrations for Colistin, were identified by broth microdilution according to the same standards. *Escherichia coli* NCTC 13846 (colistin-resistant) isolates were our quality control strains [25].

### Carbapenem resistance phenotypic and molecular detection

#### The modified carbapenem inactivation method

Briefly, a fresh bacterial suspension contained the 1-day-old colony in sterile tryptic soy broth (TSB) vortexed. Then, a 10-μg meropenem disk replaced in the suspension. It was incubated at 37 °C for 2–3 h. Then, the meropenem disk was carefully selected from the bacterial suspension and placed on an MHA plate. This plate was pre-inoculated with an *Escherichia coli* ATCC 25922 and then incubated overnight at 37 °C. The positive result for carbapenemase production was  ≤ 15 mm, the negative impact was a clear zone of  ≥ 19 mm, and the indeterminate result was 18 mm or  ≥ 19 mm. Our positive control was *K. pneumoniae* ATCC BAA-1705, and the negative control was *K. pneumoniae* ATCC BAA-1706 [25].

#### Carba NP method

Briefly, 3–5 pure colony was obtained from MHA and then dissolved in a lysis buffer. Then, add diluted phenol red solution. This solution, was prepared from 0.1 mM ZnSO4 (pH = 7.8) and 6 mg/ml imipenem. Phenol red solution without antibiotics was used as a control. All tubes shaked at 37 °C for 2 h carefully; upon hydrolysis of imipenem to a carboxyl derivative, which lowers the pH, the color of the test tube changes to a rich yellow or orange [25].

#### Molecular detection of carbapenemase resistance

The pure colonies on blood agar plates were selected for DNA extraction by boiling. Samples were incubated at 99 °C for 15 min and immediately cooled on ice. They were centrifuged at 10000 RPM for 10 min. The *blaKPC*, *blaNDM*, and *blaOXA-23* genes were amplified by specific primers listed in Table 1. Primers were designed using OLIGO 7 primer analysis software. Amplification products were observed by agarose electrophoresis 1.5%.

**Table 1:** Primers to carbapenemase producing *Klebsiella pneumoniae* by molecular method

Genes	Length of primer	Sequences	References
*blaKPC*	246 bp	5-GAT ACC ACG TTC CGT CTG G-3 5-ATT TCT GAC CGC ATT TCC AT-3	This study
*blaNDM*	621 bp	5-GGTTTGGCGATCTGGTTTTC-3 5-CGGAATGGCTCATCACGATG-3	This study
*blaOXA-23*	465 bp	5-GATCGGATTGGAGAACCAGA-3 5-ATTTCTGACCGCATTTCCAT-3	This study

### Biofilm determination of carbapenem-resistant K. pneumoniae (CRKP) isolates

The biofilm production and hypermucoviscous (HMV) phenotype are critical causes for CRKP colonization and persistence in the host. Biofilm production is important to the virulence of *K. pneumoniae* because the biofilm matrix promotes the relocation of antibiotic-resistance mobile elements while physically defending bacteria, thus enlarging microbial endurance to antibiotics, bacterial perseverance, and distribution [26–28]. Biofilm destruction needs high antimicrobial concentrations, which are often unbearable to succeed due to drug-related toxicity. Thus, regress is common even after targeted and extended therapies. According to these explanations, selecting bacterial isolates that can produce biofilm is essential for treatment [26]. Biofilm was determined using a quantitative microtiter plate (MTP) method [29]. Last, the microplate was evaluated using a microplate reader at 570 nm. The cut-off value for optical density (O.D.) was assigned. Each test was performed three times.

### Isolation, purification, and titration of the phages

The source for phage isolation was hospital wastewater. The wastewater sample was collected during the summer season at the geographical position “34.01768809 N 51.40667405 E” from the primary treatment sources of Beheshti Hospital (Kashan, Iran). The sample was collected in a 100 ml syringe and immediately transferred on ice to the microbiology department for processing. The sample was centrifuged at 10,000 × g for 15 min. The sample was filtered using a membrane with a pore size of 0.22 μm to extract the supernatant. The bacterial host for bacteriophage isolation was a Carbapenem-resistant *K. pneumoniae* (CRKP) strain isolated from a patient suffering from COVID-19 pneumoniae and resistance to Colistin with high potency for biofilm production. This isolate is called Kp100. Next, the filtered secretion mixed with an equal volume of 2 × TSB broth containing 1 ml log phase (OD600 ≈ 0.5) of the CRKP strain. This host accumulated the phages at 37 °C overnight with shaking at 180 rpm. The bacterial solution was placed in a 2-mL microtube, centrifuged at 15000 × g for 15 min, and filtered through a 0.22-μm micropore membrane to obtain a phage stock solution [30].

Then, the phage sample was serially diluted with SM buffer (5.8 g NaCl, 2.0 g MgSO4⋅7H2O, 50 ml 1 M Tris–HCl, pH 7.4, in 1 L dH2O) for phage titration by the double overlay agar plaque assay. Then, 300 μl of CRKP strain (OD600 ≈ 0.5) was mixed with a ten μl phage sample and TSB agar (0.6%). Then, the mixture was added to TSB agar (1.5%) and incubated at 37 °C for 24 h. The double-layer agar method was performed three times for each dilution. After incubation, the dilution that produced 30 to 300 plaques was selected and counted. The following formula was used to calculate the titer, and the data were expressed as a plaque-forming unit (PFU/ml) [30, 31]: Number of plaques / (dilution factor × volume of diluted phage/well) = pfu/ml.

### Phage concentration and storage

The bacteriophage stock solution was added to a 100 ml culture of CRKP strain (Kp100) in the exponential phase (1 × 10^8^ CFU/ml) at an MOI of 1 [32]. Then, 10% PEG 8000 was added. After centrifugation at 15,000 × g for 30 min at 4 °C, the pellets were resuspended in SM buffer (5.8 g NaCl, 2.0 g MgSO^4^⋅7H_2_O, 50 ml 1 M Tris–HCl, pH 7.4, in 1 L dH_2_O), and the contaminants (cell debris and endotoxins) were removed through 0.22 μm membranes and Amicon Ultra filter units (MWCO 100 kDa). After the determination of titers, the concentrated phages were stored at −80 °C until use [33].

### Identification of bacteriophage host range

The phage host range was identified using the spot test method. Briefly, 45 clinical isolates of CRKP strain and *K. pneumonia* (ATCC 10031) were included to identify the lytic spectrum of the purified isolated bacteriophage. Briefly, 150 µl (10^6^–10^8^ CFU/ml) of each overnight culture (24 h) of the CRKP strain was mixed with separately 0.8% melted agar (55 °C) and poured onto a plate covered with TSB agar. The agar was then solidified, and the filtered phage broth (10^9^ pfu/mL) was spotted onto each plate along with the above isolates. The appearance of lysis phage plaques after 8 h during the 24 h incubation period was assessed [34].

### Transmission electron microscopy (TEM) to assess phage morphology

To study the morphology of bacteriophage using a transmission electron microscope, ten microliters of the concentrated bacteriophage suspension were placed on a copper grid covered with carbon and absorbed for 5 min (excess amounts of the suspension removed carefully with filter paper). Then the grid was stained with 2% uranyl acetate for 1 min. The grid was then washed with distilled water. It was then kept in the laboratory for 1 h to completely dry. Finally, the sample was examined using a CM10Philips transmission electron microscope (Japan) at Razi Serum and Vaccination Institute (Tehran) at Kv100 voltage [35].

### The stability of phages to pH and temperature

For the pH stability of the purified phages, 10^10^ PFU/ml of the purified phage aliquots were exposed to different pH buffers (2–14) for 60 min in S.M. Buffer at 37 °C. The bilayer agar method was performed to calculate the phage titer. To determine the thermal stability of the phages, the phage solution was placed in S.M. buffer at pH 7 for 1 h at different temperatures (30 °C, 40 °C, 50 °C, 60 °C, 70 °C, and 80 °C) [34].

### Evaluation of the effect of cationic ions on phage adsorption

In this study, MgCl_2_ (final concentration of 10 Mm) and CaCl_2_ (10 mM) were used to determine cationic ions' effect on phage. These buffers were added to the culture of the phage-infected CRKP strain (Kp100). After 0, 5, 10, 15, and 20 min, the test samples were taken to determine the unabsorbed phage titer and expressed as a percentage of the original phage count.

### The optimal infection multiplication for phage identification

The optimal titer of bacteriophage was determined to reduce the concentration of the CRKP strain. A CRKP strain culture (kp100) was incubated overnight in the TSB broth medium. It grew at 37 ℃ until the OD600 of the culture reached 1.0 (1 × 10^8^ CFU/ml). A serial dilution (10^6^–10^9^ PFU/ml) of purified isolated phages was prepared at the same infection multiplicity of 0.01, 0.1, 1 and then inoculated into the fresh Kp100 culture. The mixtures were incubated at 37 ℃ and centrifuged at 15,000 g for 10 min to separate the pellet. Phosphate buffer saline (PBS) was used to wash the pellet. The bacterial mixture was serially diluted and spread on the TSB agar. The colony-forming unit (CFU/ml) is described by counting the bacteria on the plate [36].

### One‑step growth assessment

One-step growth assays were performed to identify the purified phage burst's latent cycle and size. For the test curve, 1 ml of the culture suspension of the CRKP strain (Kp100) at TSB was used. In the log phase, broth medium (OD600 = 0.1, 1 × 10^8^ CFU/ml) was combined with the purified bacteriophage with an optimal concentration of 10^6^ PFU/ml at an MOI of 0.01 and grown at 37 °C for 15 min. Phage samples were collected after 10, 20, 30, 40, 50, 60, 70, 80, 90, 100, 110, and 120 min. The number of purified phages was immediately analyzed by the bilayer agar method. All procedures repeated in three times [37]. A one-step growth curve constructed based on the number of PFU per ml. Burst size was calculated using the following formula: (titer after burst—titer at T0)/(added phage—titer at T0).

### Analysis of phage proteins under conditions of denaturation

Briefly, the denaturation loading buffer precipitated the pure bacteriophage particles. The components of the loading buffer are 0.1% bromophenol blue, 2% sodium dodecyl sulfate (SDS), 50 mM Tris–HCl, 10% glycerol, and 1% -mercaptoethanol. The heated samples were tested after 5 min in a boiling water bath at SDS-PAGE. The Coomassie Blue G-250 staining procedure produced the split protein bands.

### Extraction and analysis of the phage genome with restriction enzymes

Phage DNA was extracted using the chloroform technique; the DNA pellet was then resuspended in RNase/DNase-free water and stored at −20 °C. In this study, the manufacturer’s instructions for digestion were followed, and restriction enzymes *HindIII*, *EcoRI*, *PaeI*, *SspI*, and *NdeI* from Sigma Aldrich were used. These enzymes performed this restriction digestion process three times. Finally, this study used 1% TBE (Tris–Borate EDTA) running buffer and 1% agarose gel electrophoresis to study our digestion.

### Whole phage genome and annotation

The whole genome of lytic phage was sequenced using Illumina HiSeq technology (Illumina, San Diego, CA). The genome sequencing quality was checked for completeness and contamination using a CheckV [38]. For quality assurance and preprocessing, such as trimming, Geneious Prime 2021.2.2 (https://www.geneious.com) and Spades 3.9.0 with 580-fold sequence coverage performed de novo assembly of the raw sequencing data. The whole genome sequence of the lytic phage was linked to the GenBank database accession number. The RASTtk—v1.073 and PHASTER software (https://phaster.ca/) were used to predict and annotate the ORFs [39]. ResFinder is used to assess the presence of antimicrobial resistance and virulence genes in the phage genome [40]. The tRNAs can-SE v2.0.6 is used to determine the presence of tRNA in the phage genome [41]. Linear genome comparisons of phages and visualization of coding regions were performed using Easyfig 2.2.3 [42] and Mauve 2.4.0 [43]. Phage lifestyles were predicted using the program PHACTS [44]. Sequence similarity was determined for the subsequent bioinformatics study using BLASTp searches in the NCBI database (http://www.ncbi.nlm.nih.gov/BLAST).

### Phylogenetic analysis of the phage

Phylogenetic analysis and amino acid sequences of phage core proteins, such as the major capsid protein, were checked for similarities in the NCBI database using the BLASTp tool. In addition, phages with homology to the amino acid sequences of these phage proteins were selected to generate phylogenetic trees using molecular evolutionary genetics analysis [45] software version 11.0. Phylogenies were reconstructed using the Neighbor-Joining method with 1000 bootstrap repeats [46]. HHpred software was used to predict depolymerase (https://toolkit.tuebingen.mpg.de/tools/hhpred) [47].

### Methods of statistical analysis

In this study, GraphPad Prism version 9.1 was used for the statistical analysis. Student’s t-test was used for the comparison between the sets and the control sets, and the significance level was set at *P ≤ 0.05*.

## Results

### Bacterial isolation, identification, antibiotic susceptibility test, and biofilm determination

**Fig. 1:**
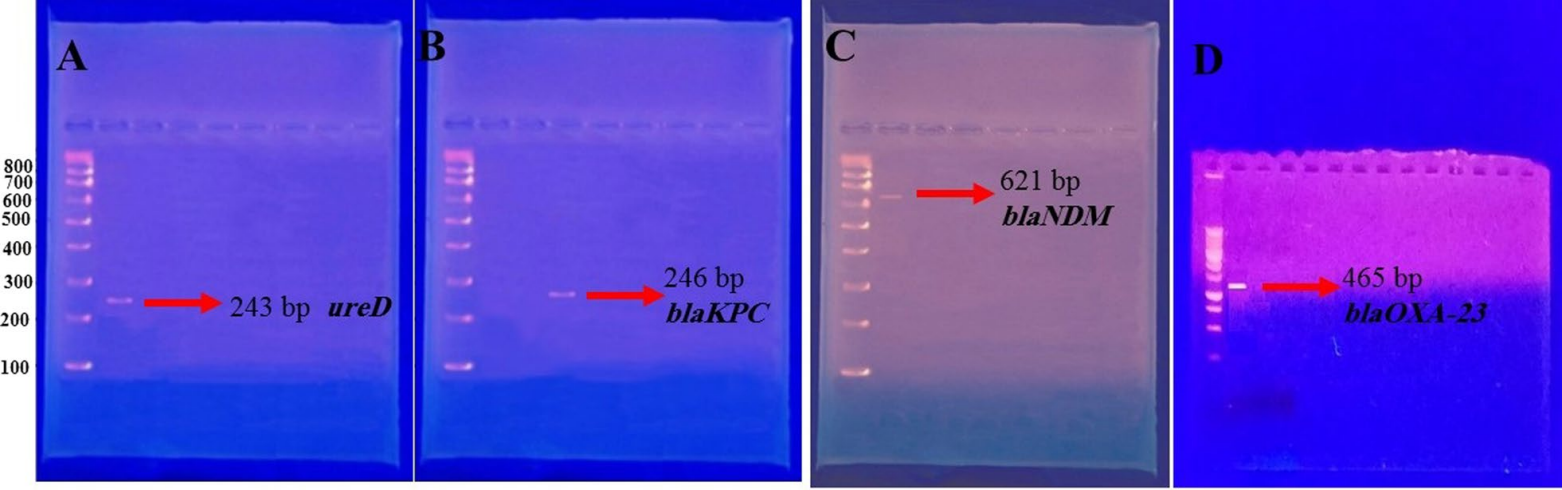
Electrophoresis pattern of *K. pneumoniae* confirmation genes by *a ureD* gene with 243 bp fragment **a** c and carbapenemase confirmation genes *blaKPC* (**b**), *blaNDM* (**c**) and *blaOXA-23* (**d**)

**Fig. 2:**
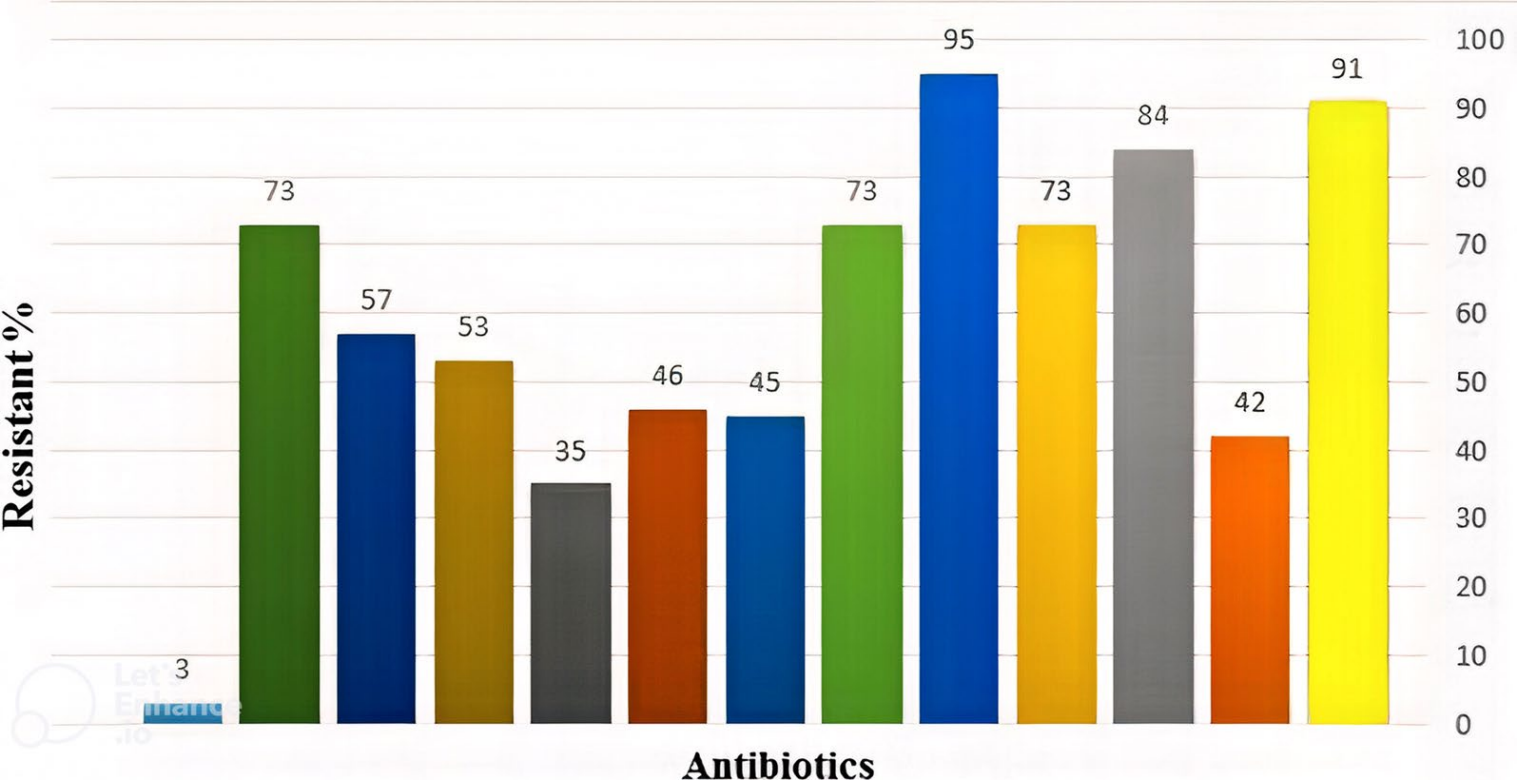
Frequency distribution of antibiotic resistance in clinical samples according to the type of antibiotic

One hundred isolates of *K. pneumoniae* were identified by phenotypic and biochemical approaches from all 103 tracheal samples (97.7%). PCR confirmed the positive samples for the *ureD* gene (243 bp) (Fig. 1). Forty-five isolates were carbapenemase-producing in this study; the highest resistance rate was associated with 97 isolates (95.1%) with ciprofloxacin, and the most heightened susceptibility was associated with 99 isolates (97.1%) with Colistin (Fig. 2).

**Fig. 3:**
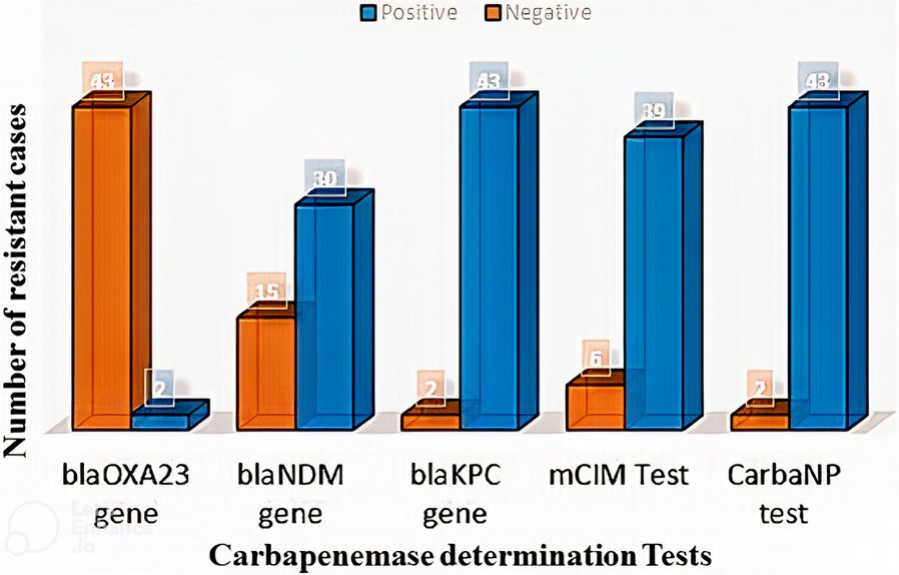
Frequency distribution of phenotypic and molecular test results to determine Carbapenem-resistant *K. pneumoniae* (CRKP) clinical isolates

All 45 isolates were classified in the carbapenem-resistant *K. pneumoniae* (CRKP) group. For the carbapenemase-producing phenotype, the scores were determined by the CarbaNP assay (95.6%) and the mCIM assay (86.7%). On molecular analysis, the presence of the *blaKPC* gene was 95.6%, *the blaNDM gene* was 66.7%, and the least amount was for the *blaOXA 23* gene (4.4%) (Fig. 3).

Most of the 100 isolates of *K. pneumoniae* (66%) were negative for biofilm production ability. Among all carbapenemase-producing isolates, the ability to produce biofilm was highest with medium strength (42%). The ability to produce at weak strength was (9%), and the ability to produce at high strength was (11%) (Table 2).

**Table 2:** Characteristics of clinical *Klebsiella pneumoniae* isolates

Number	*K.p* isolated codes	The Resistance pattern of antibiotics	*Klebsiella pneumoniae* carbapenemase (KPC) isolates	Carba NP test	mCIM test	BlaKPC gene	BlaNDM gene	blaOXA-23 gene	Colistin MIC interpretation	Biofilm formation potency
1	Kp1	AMC, SXT, CTX, TOB, AZT, GM, CIP, PTZ	−	N	N	−	−	−	Not applicable	Negative
2	Kp2	AMC, SXT, MER, CPM, TOB, AZT, IMI, CIP, GM, CTX, PTZ	+	P	P	+	−	−	Sensitive	Weakly
3	Kp3	AMC, SXT, MER, CPM, TOB, AZT, IMI, CIP, GM, CTX, PTZ	+	P	P	+	+	−	Sensitive	Negative
4	Kp4	AMC, SXT, MER, CPM, TOB, AZT, IMI, CIP, GM, CTX, PTZ	+	P	P	+	+	−	Sensitive	Negative
5	Kp5	Non-resistance	−	N	N	−	−	−	Not applicable	Negative
6	Kp6	AMC, SXT, CPM, CTX, TOB, AZT, GM, CIP, PTZ	−	N	N	−	−	−	Not applicable	Negative
7	Kp7	SXT, GM, CIP, PTZ	−	N	N	−	−	−	Not applicable	Negative
8	Kp8	AMC, SXT, CPM, CTX, TOB, AZT, GM, CIP, PTZ	−	N	N	−	−	−	Not applicable	Negative
9	Kp9	AMC, SXT, CPM, CTX, TOB, AZT, GM, CIP, PTZ	−	N	N	−	−	−	Not applicable	Negative
10	Kp10	AMC, SXT, MER, CPM, TOB, AZT, IMI, GEM, ETP, CIP, CTX, PTZ	+	P	P	+	+	−	Sensitive	Moderate
11	Kp11	AMC, SXT, MER, CPM, TOB, AZT, IMI, GEM, ETP, CIP, CTX, PTZ	+	N	N	+	−	−	Sensitive	Moderate
12	Kp12	AMC, SXT, CPM, CTX, TOB, AZT, GM, CIP, PTZ	−	N	N	−	−	−	Not applicable	Negative
13	Kp13	AMC, CPM, AZT, GM, CIP, PTZ	−	N	N	−	−	−	Not applicable	Negative
14	Kp14	AMC, SXT, MER, CPM, TOB, AZT, IMI, CIP, GM, CTX, PTZ	+	P	P	+	−	−	Sensitive	Negative
15	Kp15	SXT, MER, CPM, CTX, AZT, IMI, ETP, GM, CIP, PTZ	+	P	N	+	−	−	Sensitive	Weakly
16	Kp16	SXT, CIP	−	N	N	−	−	−	Not applicable	Negative
17	Kp17	Non-resistance	−	N	N	−	−	−	Not applicable	Negative
18	Kp18	SXT, CPM, AZT, GM, CIP, CTX, PTZ	−	N	N	−	−	−	Not applicable	Negative
19	Kp19	SXT, CPM, AZT, GM, CIP, CTX, PTZ	−	N	N	−	−	−	Not applicable	Negative
20	Kp20	Non-resistance	−	N	N	−	−	−	Not applicable	Negative
21	Kp21	AMC, SXT, CIP, GM	−	N	N	−	−	−	Not applicable	Negative
22	Kp22	SXT, CPM, AZT, GM, CIP, CTX, PTZ	−	N	N	−	−	−	Not applicable	Moderate
23	Kp23	AMC, SXT, MER, CPM, TOB, AZT, IMI, GEM, ETP, CIP, CTX, PTZ	+	P	P	+	+	+	Sensitive	Moderate
24	Kp24	SXT, CIP	−	N	N	−	−	−	Not applicable	Negative
25	Kp25	Non-resistance	−	N	N	−	−	−	Not applicable	Negative
26	Kp26	AMC, SXT, CIP, GM	−	N	N	−	−	−	Not applicable	Negative
27	Kp27	AMC, SXT, MER, CPM, TOB, AZT, IMI, CIP, GM, CTX, PTZ	+	P	P	+	+	−	Sensitive	Negative
28	Kp28	AMC, SXT, MER, CPM, TOB, AZT, IMI, GEM, ETP, CIP, CTX, PTZ	+	P	P	+	+	−	Sensitive	Moderate
29	Kp29	SXT, CPM, AZT, GM, CIP, CTX, PTZ	−	N	N	−	−	−	Not applicable	Negative
30	Kp30	SXT, CPM, AZT, GM, CIP, CTX, PTZ	−	N	N	−	−	−	Not applicable	Negative
31	Kp31	SXT, TOB, CIP, PTZ	−	N	N	−	−	−	Not applicable	Negative
32	Kp32	AMC, SXT, MER, CPM, TOB, AZT, IMI, CIP, GM, CTX, PTZ	+	P	P	+	−	−	Sensitive	Negative
33	Kp33	SXT, CIP	−	N	N	−	−	−	Not applicable	Negative
34	Kp34	AMC, SXT, MER, CPM, TOB, AZT, IMI, CIP, GM, CTX, PTZ	+	P	P	+	+	−	Sensitive	Moderate
35	Kp35	AMC, SXT, MER, CPM, TOB, AZT, IMI, GEM, ETP, CIP, CTX, PTZ	+	P	P	+	+	−	Sensitive	Strong
36	Kp36	SXT, MER, CPM, CTX, AZT, IMI, ETP, GM, CIP, PTZ	+	P	N	+	+	−	Sensitive	Moderate
37	Kp37	AMC, SXT, MER, CPM, TOB, AZT, IMI, GEM, ETP, CIP, CTX, PTZ	+	N	N	+	+	−	Sensitive	Negative
38	Kp38	SXT, CPM, AZT, GM, CIP, CTX, PTZ	−	N	N	−	−	−	Not applicable	Moderate
39	Kp39	SXT, CPM, AZT, GM, CIP, CTX, PTZ	−	N	N	−	−	−	Not applicable	Moderate
40	Kp40	SXT, CPM, AZT, GM, CIP, CTX, PTZ	−	N	N	−	−	−	Not applicable	Weakly
41	Kp41	AMC, SXT, MER, CPM, TOB, AZT, IMI, GEM, ETP, CIP, CTX, PTZ	+	P	P	+	+	-	Sensitive	Moderate
42	Kp42	SXT, CIP	−	N	N	−	−	−	Not applicable	Negative
43	Kp43	SXT, MER, CPM, CTX, AZT, IMI, ETP, GM, CIP, PTZ	+	P	P	+	+	−	Sensitive	Negative
44	Kp44	AMC, SXT, MER, CPM, TOB, AZT, IMI, GEM, ETP, CIP, CTX, PTZ	+	P	P	−	+	−	Sensitive	Moderate
45	Kp45	SXT, TOB, CIP, PTZ	−	N	N	−	−	−	Not applicable	Negative
46	Kp46	AMC, SXT, MER, CPM, TOB, AZT, IMI, GEM, ETP, CIP, CTX, PTZ	+	P	P	+	−	−	Sensitive	Moderate
47	Kp47	AMC, SXT, MER, CPM, TOB, AZT, IMI, GEM, ETP, CIP, CTX, PTZ	+	P	P	+	+	−	Sensitive	Moderate
48	Kp48	AMC, SXT, MER, CPM, TOB, AZT, IMI, GEM, ETP, CIP, CTX, PTZ	+	P	P	+	+	−	Sensitive	Moderate
49	Kp49	SXT, MER, CPM, CTX, AZT, IMI, ETP, GM, CIP, PTZ	+	P	P	+	+	−	Sensitive	Negative
50	Kp50	SXT, MER, CPM, CTX, AZT, IMI, ETP, GM, CIP, PTZ	+	P	N	+	−	−	Sensitive	Negative
51	Kp51	SXT, CPM, AZT, GM, CIP, CTX, PTZ	−	N	N	−	−	−	Not applicable	Negative
52	Kp52	AMC, SXT, CIP, GM	−	N	N	−	−	−	Not applicable	Negative
53	Kp53	SXT, TOB, CIP, PTZ	−	N	N	−	−	−	Not applicable	Negative
54	Kp54	AMC, SXT, MER, CPM, TOB, AZT, IMI, CIP, GM, CTX, PTZ	+	P	P	+	+	−	Sensitive	Negative
55	Kp55	SXT, TOB, CIP	−	N	N	−	−	−	Not applicable	Negative
56	Kp56	CPM, TOB, AZT, CIP, PTZ	−	N	N	−	−	−	Not applicable	Negative
57	Kp57	MER, TOB, CIP	−	N	N	−	−	−	Not applicable	Negative
58	Kp58	MER, CPM, TOB, AZT, IMI, GM, CIP, PTZ	+	P	P	+	+	−	Sensitive	Negative
59	Kp59	AMC, SXT, MER, CPM, TOB, AZT, IMI, GEM, ETP, CIP, CTX, PTZ	+	P	P	+	+	−	Sensitive	Moderate
60	Kp60	SXT, TOB, CIP, PTZ	−	N	N	−	−	−	Not applicable	Negative
61	Kp61	SXT, CPM, AZT, GM, CIP, CTX, PTZ	−	N	N	−	−	-	Not applicable	Negative
62	Kp62	SXT, MER, CPM, CTX, AZT, IMI, ETP, GM, CIP, PTZ	+	P	P	+	−	−	Sensitive	Negative
63	Kp63	SXT, TOB, CIP, PTZ	−	N	N	−	−	−	Not applicable	Negative
64	Kp64	AMC, SXT, MER, CPM, TOB, AZT, IMI, GEM, ETP, CIP, CTX, PTZ	+	P	P	+	+	−	Sensitive	Strong
65	Kp65	SXT, CPM, AZT, GM, CIP, CTX, PT	−	N	N	−	−	−	Not applicable	Weak
66	Kp66	SXT, CPM, TOB, AZT, CTX, GM, CIP, PTZ	−	N	N	−	−	−	Not applicable	Negative
67	Kp67	SXT, CPM, TOB, AZT, CTX, GM, CIP, PTZ	−	N	N	−	−	−	Not applicable	Negative
68	Kp68	SXT, CPM, TOB, AZT, CTX, GM, CIP, PTZ	−	N	N	−	−	−	Not applicable	Negative
69	Kp69	SXT, MER, CPM, CTX, AZT, IMI, ETP, GM, CIP, PTZ	+	P	P	+	+	−	Sensitive	Negative
70	Kp70	SXT, TOB, CIP, PTZ	−	N	P	−	−	−	Not applicable	Negative
71	Kp71	AMC, SXT, CIP, GM	−	N	N	−	−	−	Not applicable	Negative
72	Kp72	AMC, SXT, MER, CPM, TOB, AZT, IMI, GEM, ETP, CIP, CTX, PTZ	+	P	P	+	+	−	Sensitive	Moderate
73	Kp73	SXT, MER, CPM, CTX, AZT, IMI, ETP, GM, CIP, PTZ	+	P	P	+	−	−	Sensitive	Negative
74	Kp74	SXT, MER, CPM, CTX, AZT, IMI, ETP, GM, CIP, PTZ	+	P	P	+	−	−	Sensitive	Negative
75	Kp75	SXT, MER, CPM, CTX, AZT, IMI, ETP, GM, CIP, PTZ	+	P	P	+	+	−	Sensitive	Negative
76	Kp76	SXT, CPM, AZT, GM, CIP, CTX, PTZ	−	N	N	−	−	−	Not applicable	Negative
77	Kp77	AMC, SXT, MER, CPM, TOB, AZT, IMI, CIP, GM, CTX, PTZ	+	P	N	+	−	−	Sensitive	Moderate
78	Kp78	AMC, SXT, CIP, GM	−	N	N	−	−	−	Not applicable	Negative
79	Kp79	Non-resistance	−	N	N	−	−	−	Not applicable	Negative
80	Kp80	SXT, MER, CPM, CTX, AZT, IMI, ETP, GM, CIP, PTZ	+	P	P	+	−	−	Sensitive	Weakly
81	Kp81	AMC, SXT, CIP, GM	−	N	N	−	−	−	Not applicable	Negative
82	Kp82	SXT, MER, CPM, CTX, AZT, IMI, ETP, GM, CIP, PTZ	+	P	P	+	−	−	Sensitive	Weakly
83	Kp83	SXT, PTZ, CIP	−	N	N	−	−	−	Not applicable	Negative
84	Kp84	SXT, CPM, TOB, AZT, CTX, GM, CIP, PTZ	−	N	N	−	−	−	Not applicable	Negative
85	Kp85	SXT, MER, CPM, CTX, AZT, IMI, ETP, GM, CIP, PTZ	+	P	P	+	+	−	Sensitive	Moderate
86	Kp86	SXT, CPM, TOB, AZT, CTX, GM, CIP, PTZ	−	N	N	−	−	−	Not applicable	Negative
87	Kp87	SXT, CPM, TOB, AZT, CTX, GM, CIP, PTZ	−	N	N	−	−	−	Not applicable	Negative
88	Kp88	SXT, PTZ, CIP	−	N	N	−	−	−	Not applicable	Negative
89	Kp89	SXT, CPM, AZT, GM, CIP, CTX, PTZ	−	N	N	−	−	−	Not applicable	Negative
90	Kp90	AMC, SXT, MER, CPM, TOB, AZT, IMI, GEM, ETP, CIP, CTX, PTZ	+	P	P	+	−	−	Sensitive	Moderate
91	Kp91	AMC, SXT, MER, CPM, TOB, AZT, IMI, GEM, ETP, CIP, CTX, PTZ	+	P	P	+	+	−	Sensitive	Moderate
92	Kp92	SXT, CPM, TOB, AZT, CTX, GM, CIP, PTZ	−	N	N	−	+	−	Not applicable	Negative
93	Kp93	SXT, PTZ, CIP	−	N	N	−	−	−	Not applicable	Negative
94	Kp94	SXT, MER, CPM, CTX, AZT, IMI, ETP, GM, CIP, PTZ	+	P	P	+	−	−	Sensitive	Negative
95	Kp95	AMC, SXT, MER, CPM, TOB, AZT, IMI, GEM, ETP, CIP, CTX, PTZ	+	P	P	+	+	−	Sensitive	Strong
96	Kp96	SXT, CPM, TOB, AZT, CTX, GM, CIP, PTZ	−	N	N	−	+	−	Not applicable	Negative
97	Kp97	AMC, SXT, MER, CPM, TOB, AZT, IMI, GEM, ETP, CIP, CTX, PT	+	P	P	+	+	+	Sensitive	Strong
98	Kp98	AMC, SXT, MER, CPM, TOB, AZT, IMI, GEM, ETP, CIP, CTX, PTZ	+	P	P	+	+	−	Resistance	Moderate
99	Kp99	AMC, SXT, MER, CPM, TOB, AZT, IMI, GEM, ETP, CIP, CTX, PTZ	+	P	P	+	+	−	Resistance	Moderate
100	Kp100	AMC, SXT, MER, CPM, TOB, AZT, IMI, GEM, ETP, CIP, CTX, PTZ	+	P	P	+	+	+	Resistance	Strong

Trimethoprim/sulfamethoxazole (1.25/23.75 μg) (SXT), Amoxicillin-clavulanate (20/10 μg) (AMC), piperacillin/tazobactam (100/10 μg) (PTZ), Cefepime (30 μg) (CPM), Cefotaxime (30 μg) (CTX), Ciprofloxacin (5 μg) (CIP), Imipenem (10 μg) (IMI), Ertapenem (10 μg) (ETP), Meropenem (10 μg) (MER), Tobramycin (10 μg) (TOB), Gentamicin (10 μg) (GM), Aztreonam (30 μg) (AZT). Kp: *Klebsiella pneumoniae*, *P* positive, *N* Negative

### VB_KshKPC-M phage plaque and virion morphology

**Fig. 4:**
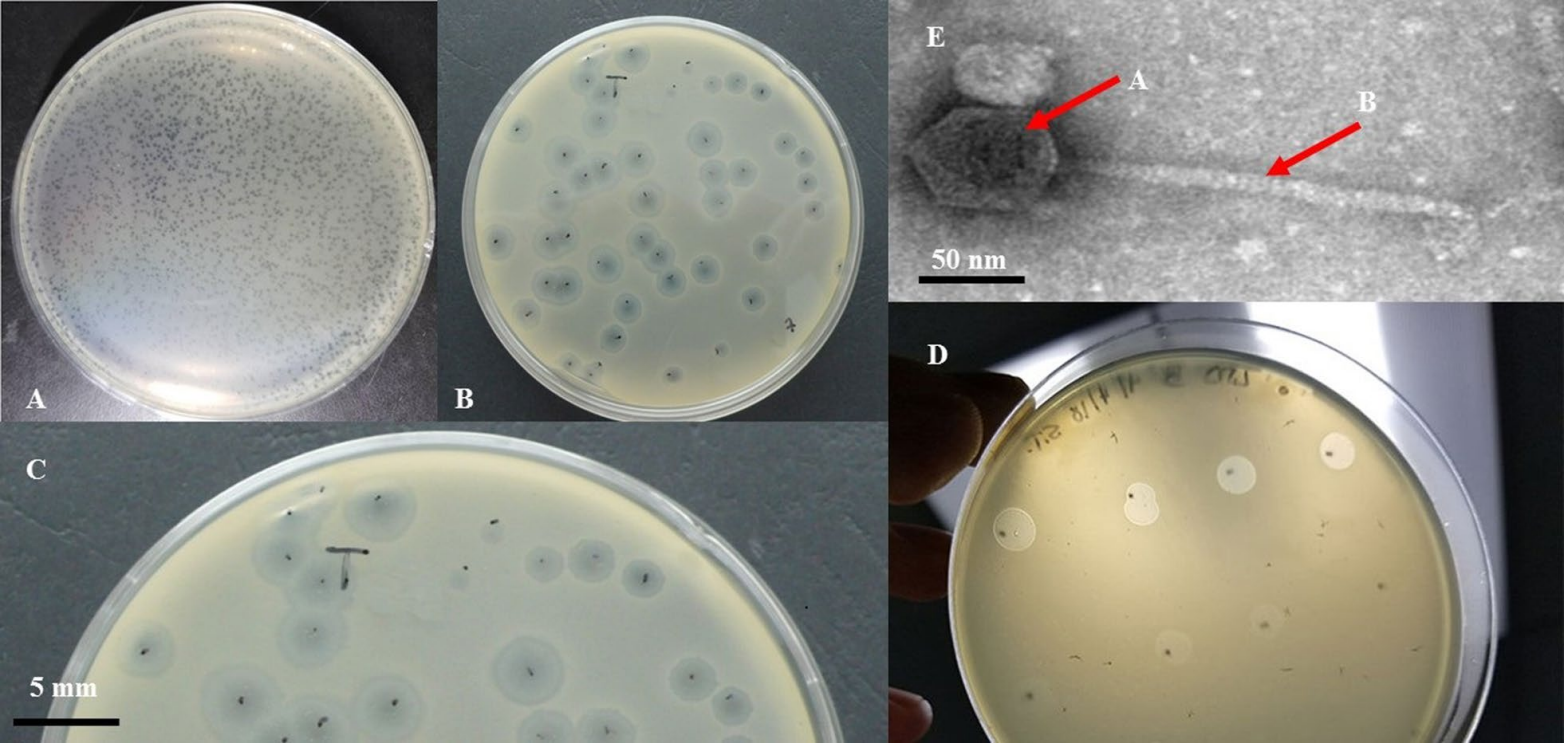
The presence of bacteriophage in the studied sample. **A, B, and C**: plaque morphology of vB_KshKPC-M. Phages were cultured on the Kp100 strain (Carbapenemase resistance *K. pneumoniae*), forming 4 mm plaques (4 ± 0.5 mm in diameter) with a clear center surrounded by a halo. **D**: titration of vB_KshKPC-M phage. **E**: Transmission electron microscope micrograph of negatively stained phage vB_KshKPC-M at 30,000 × magnification. vB_KshKPC-M has (**A**) a head diameter of approximately 50 nm (50 ± 5 nm) and (**B**) a flexible non-contractile tail approximately 145 nm long (145 ± 5 nm)

VB_KshKPC-M forms transparent, circular plaques with halos approximately 4 mm in diameter (4 ± 0.5 mm) on a lawn of its host bacterium Kp100 (a carbapenem-resistant *K. pneumoniae* (CRKP) isolate) (Fig. 4A, B, C).

TEM confirms that VB_KshKPC-M is a phage belonging to the order *Caudovirales* with a head diameter of 50 nm (50 ± 5 nm) and a flexible, non-contractile tail of 145 nm (145 ± 5 nm) (Fig. 2E). This type of phage was previously classified as a member of the *Siphoviridae* family according to the most recent recommendations of the ICTV (International Committee on Taxonomy of Viruses, http://ictv.global/taxonomyRelease.s.asp). However, since the introduction of new phage families, phages can no longer be assigned to a specific family based on micrographs alone. The VB_KshKPC-M is comparable in head-to-tail proportions (the head diameter is approximately 35% of the tail length; Fig. 2E) to the T1-like phage vB_EcoS_ACG-M12 [48].

### Host range of the phage VB_KshKPC-M

The strain used for phage isolation was one of 45 strains used to investigate the lytic range of VB_KshKPC-M. As shown in Table 3, the clinical CRKP isolates that had plaques of different sizes on the lawn plate were lysed by the phage VB_KshKPC-M in 97.7% (44/45) of the cases. Kp15, a specific CRKP strain, proved immune to the phage.

**Table 3:** Host range of plaques *VB_KshKPC-M*

Carbapenem-resistant *Klebsiella pneumoniae* (CRKP) strains	Lytic spots of phage
Kp2	+
Kp3	+
Kp4	+
Kp10	+
Kp11	+
Kp14	+
Kp15	−
Kp23	+
Kp27	+
Kp28	+
Kp32	+
Kp34	+
Kp35	+
Kp36	+
Kp37	+
Kp41	+
Kp43	+
Kp44	+
Kp46	+
Kp47	+
Kp48	+
Kp49	+
Kp50	+
Kp54	+
Kp58	+
Kp59	+
Kp62	+
Kp64	+
Kp69	+
Kp72	+
Kp73	+
Kp74	+
Kp75	+
Kp77	+
Kp80	+
Kp82	+
Kp85	+
Kp90	+
Kp91	+
Kp94	+
Kp95	+
Kp97	+
Kp98	+
Kp99	+
Kp100	+

### Stability of bacteriophage

**Fig. 5:**
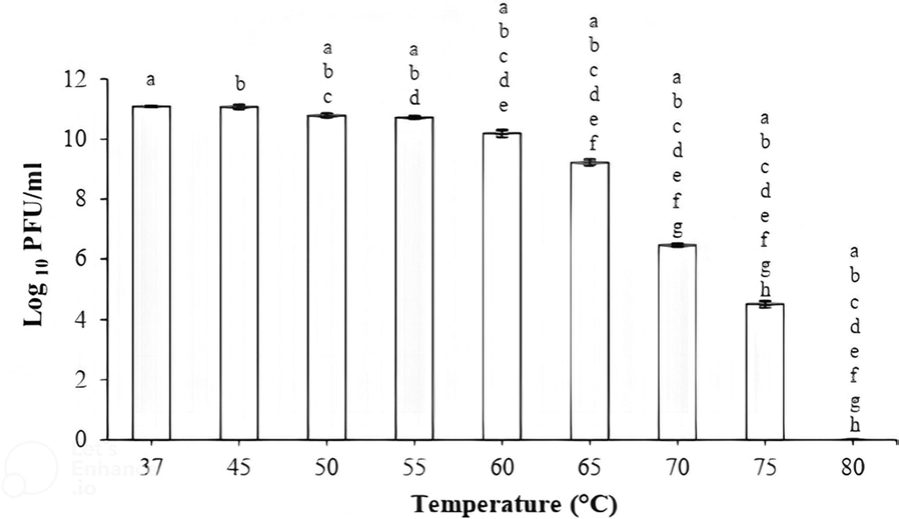
Temperature stability of phage vB_KshKPC-M at different temperatures for 1 h. Similar letters on each of the columns indicate a significant difference in phage titer at different temperatures (One-way ANOVA Repeated measures, *P* ≤ *0.05*)

The results of the temperature stability of bacteriophage showed that phage vB_KshKPC-M was most active after 1 h of incubation in the temperature range of 37 to 45 °C, so there was no statistically significant difference between these two temperatures. However, there was a significant difference at different temperatures. (One-way ANOVA Repeated measures, *P* ≤ *0.05*). As the temperature increased, the bacteriophage activity decreased, so the phages were deactivated entirely after 1 h of incubation at 80, 75, and 70 °C, respectively (Fig. 5).

**Fig. 6:**
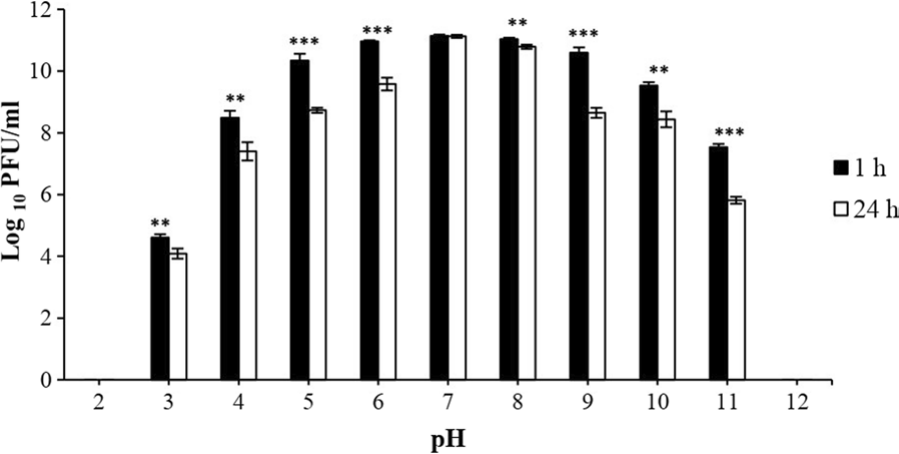
Stability of phage vB_KshKPC-M at different pH after 1 and 24 h of incubation at 37 °C. The star indicated a significant level in the phage titer after 1 h of treatment at different pH compared to 24 h of incubation. (T-test: P* ≤ 0.05, P** ≤ 0.01, P*** ≤ 0.001)

The stability of bacteriophage at different pH values for 1 h and 24 h at 37 °C showed that phage vB_KshKPC-M was most active at pH seven after 1 h and 24 h of incubation; the phage titer at this pH was significantly different. It was no more active after 24 h than after 1 h. At other pH values (3–11), phage stability decreased considerably after 24 h compared with 1 h of incubation at 37 °C (T-test P ≤ 0.05) graph (Fig. 6).

### Cationic ions and adsorption rate of phage

**Fig. 7:**
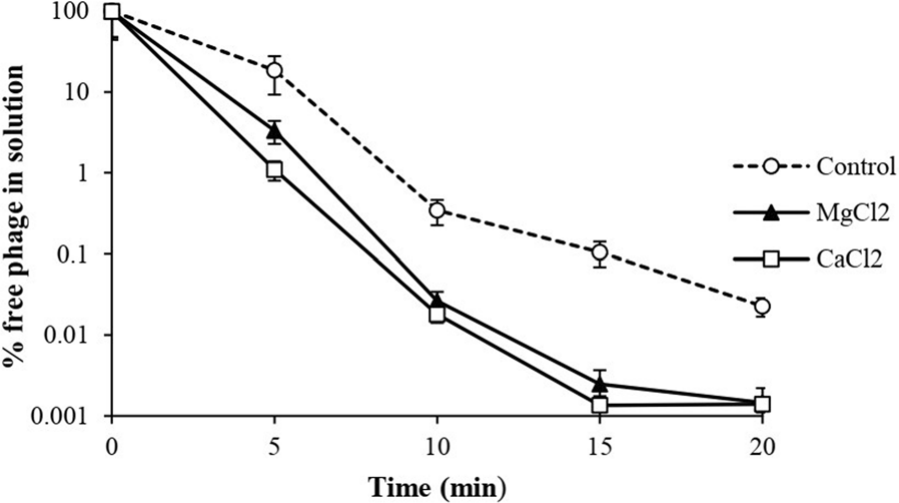
Assessment of divalent ions MgCl_2_ and CaCl_2_ on the amount of vB_KshKPC-M phage adsorption to CRKP strain, Kp100 host

The results of the evaluation of the influence of divalent cations on the adsorption rate of phage to host bacteria were presented as follows:

The rate of absorption of vB_KshKPC-M phage to its host in the control sample (without metal ions) was 81.5% after 5 min. At the same time, this rate was 98.9% and 96.7% in the presence of calcium and magnesium ions, respectively. The maximum rate of phage absorption into the host occurred after 20 min. It was 99.98% in the control mixture and 99.998% in the combination treated with the divalent cation (Fig. 7). Statistical analysis showed a significant difference between the amount of phage absorption in the control groups (phage mixture with specific host bacteria) and the phage treated with calcium or magnesium. (One-way ANOVA Repeated measures, *P* ≤ *0.05*).

### Evaluation of one-step growth

**Fig. 8:**
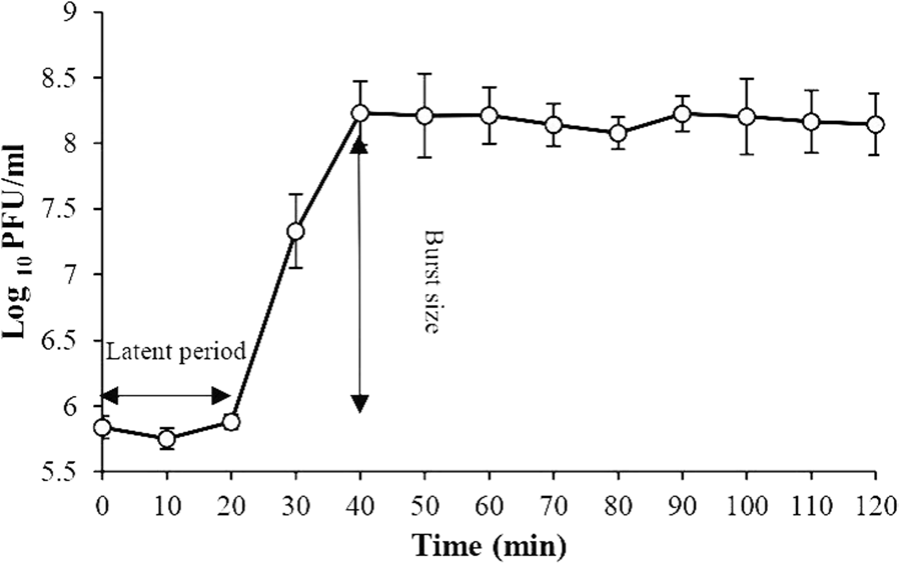
The one-step growth curve of phage vB_KshKPC-M was drawn according to incubation time and progeny production rate

The growth curve was drawn. Based on this, the latency time (time interval between uptake of phage into bacterial cell and release of the first phage produced), the constant titer, and production rate of phage progeny were calculated. The growth curve of phage vB_KshKPC-M relative to the Kp100 strain (a CRKP strain) is shown in Fig. 8. Phage vB_KshKPC-M has a relatively short incubation period, lasting about 20 min, and its burst size is about 260 phage particles per infected cell.

### Restriction analysis of phage DNA

**Fig. 9:**
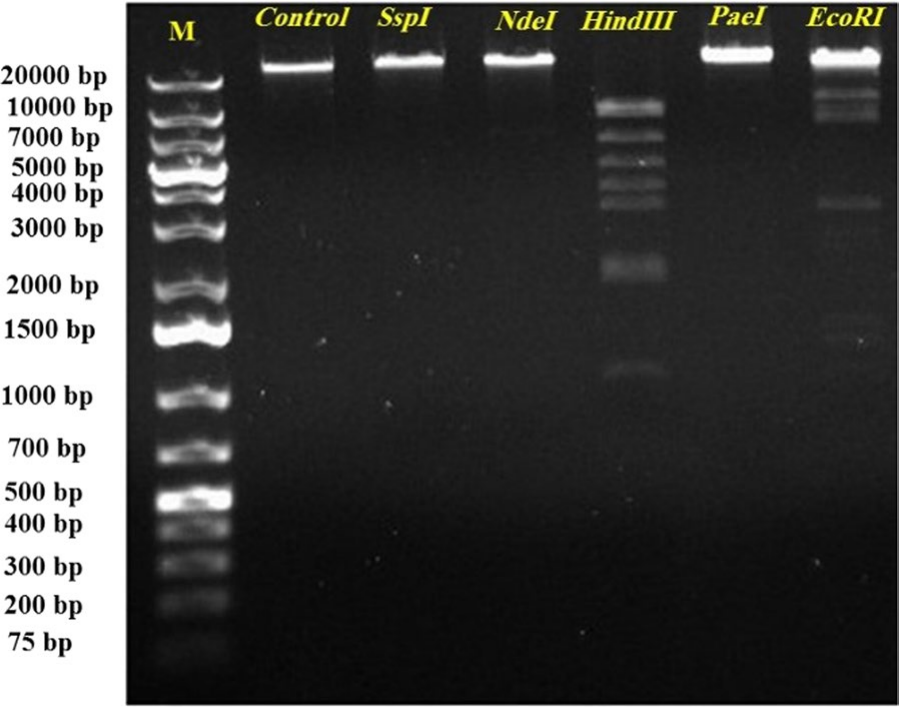
The results of enzymatic digestion of bacteriophage vB_KshKPC-M genome by restriction enzymes to determine the cutting pattern and estimate the genome size. M: kb 25 markers (Sina clone, Iran)

The results showed that the genome of bacteriophage vB_KshKPC-M was sensitive to only *EcoRI* and *HindIII* among the restriction enzymes *EcoR I*, *Ssp I*, *Nde I*, *Pae I*, and *HindIII* (Fig. 9). From the enzymatic digestion of the phage genome by the endonucleases above, it was concluded that the bacteriophage genome was double-stranded. Based on the results obtained from the size of the cut genome fragments using the SequentiX Gel Analyzer software, the approximate size of the bacteriophage genome was estimated to be about 50 Kb.

### Analysis of phage proteins under conditions of denaturation

**Fig. 10:**
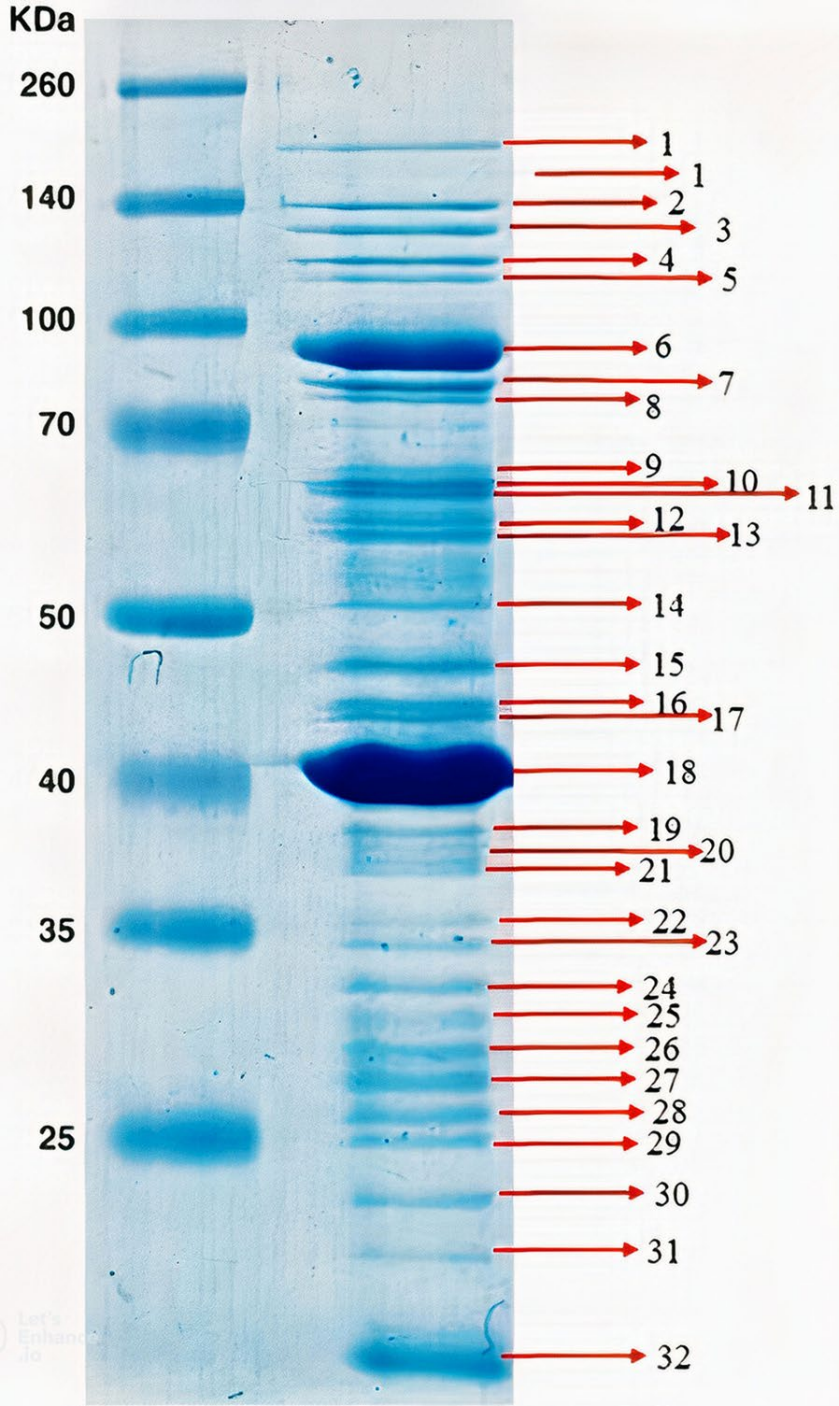
SDS-PAGE pattern of isolated bacteriophage proteins. 245 kDa protein marker (Sina Clone, Iran)

The vB_KshKPC-M phage comprises at least 32 proteins, according to protein analysis. Two significant proteins have 40 and 90 kDa weights and at least 30 minor polypeptides with molecular weights ranging from 2 to 250 kDa (Fig. 10).

### Killing dynamics of vB_KshKPC-M against CRKP strain Kp100

**Fig. 11:**
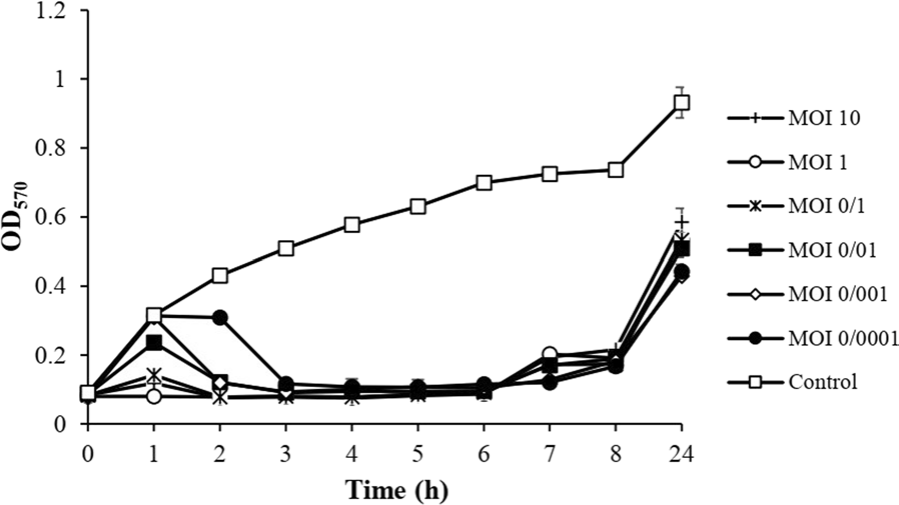
Measuring the optical absorbance of CRKP strain, Kp100 treated with different MOIs of phage vB_KshKPC-M at a wavelength of 570 nm during 24 h of incubation

The effect of different MOI of phage vB_KshKPC-M on CRKP strain, Kp100, is shown in Fig. 11. The graph shows that phages with higher MOIs (1, 10, and 0.1) have similar absorption curves at different times. And also, for lower MOIs (0.001, 0.01, and 0.0001), the absorbance curve is approximately the same at other times. Between 3 and 6 h, the absorption curve was almost the same for all MOI, and it seems that the phage has its most potent effect at these hours. After 6 h, the amount of absorption increased for all MOIs. After 24 h, the amount of absorption was slightly lower in the wells treated with lower MOI (0.001, 0.01, and 0.0001) (OD = 570 0.4) compared to higher MOI (1, 10 and 0.1) (OD = 0.5) while these results were opposite in the first hours (1 to 3). Based on the obtained results, two high and low dilutions of the phage MOI (1 and 0.001) were selected for further experiments (Fig. 11).

### Whole genome sequencing analysis and ORF prediction of vB_KshKPC-M

Bioinformatics analysis of the vB_KshKPC-M phage genome showed that the genome of this bacteriophage was a double-stranded linear DNA consisting of 54378 base pairs with 50.8% guanine + cytosine. The genome contained 91 putative ORF; most (52.74%, 48 ORFs) were on the negative or complementary strand. Eighty-seven ORFs (95.6%) with an ATG start codon, three ORFs (3.3%), including ORF46, ORF64, and ORF89 with a GTG start codon, and one (ORF66) ORF with a TTG start codon. Three types of termination codons were identified for putative ORFs, which include: TAA as the most common termination codon (54.9%, 50 (ORF, TGA (31.9%, 29 ORF), and TAG (13.2%, 12 ORF). The results of detecting the presence of tRNA showed that the genome sequence of phage vB_KshKPC-M did not contain any tRNA genes.

**Fig. 12:**
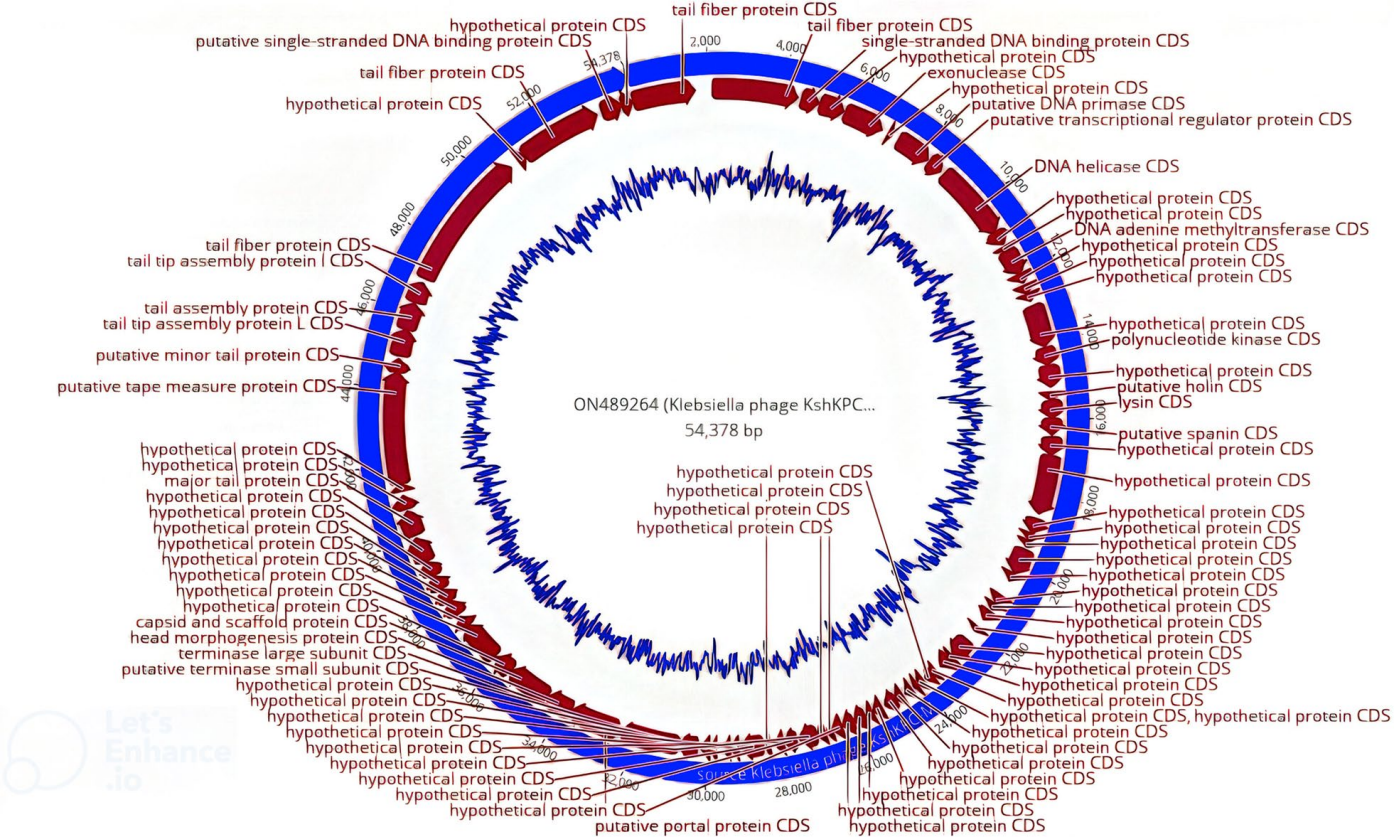
Genetic image of phage vB_KshKPC-M using GeniousPrime 2022.2.2

**Fig. 13:**
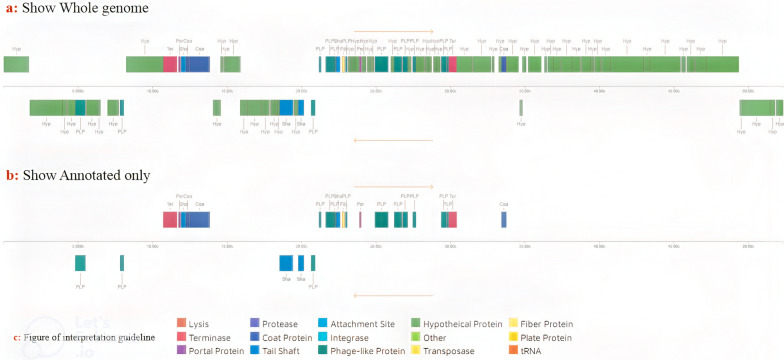
Genome analysis of vB_KshKPC-M phage using PHASTER software (https://phaster.ca/), the number of predicted ORFs, and their functional characteristics are shown in the figure with different colors. **a**: determine the whole genome diagram with no tRNA genes (Orange color in the guideline), **b**: show annotated only diagram (with no tRNA genes), and **c**: color interpretation guideline. The orange color related to tRNA, which revealed no tRNA genes determined

Of the total 84 hypothetical ORFs, 23 ORFs have low similarity to specific functional genes recorded in Genebank. Thirty-three ORFs are identical to hypothetical proteins with an unclear function registered in the Genebank. The remaining twenty-eight ORFs were not similar to any of the sequences in the gene bank. BLAST results for the predicted ORFs were obtained. Based on the obtained results, the genetic map of phage vB_KshKPC-M was constructed using GeniousPrime 2022.2.2 (Fig. 12). The function of all predicted ORFs of phage vB_KshKPC-M was also obtained using PHAST software (Fig. 13). The results agreed well and were similar to those obtained from the analysis of BLAST.

### Phylogenetic and comparative genomic analysis

The complete nucleotide sequence (BLASTn) of phage vB_KshKPC-M in Genebank, optimized for sequences with high similarity (Megablast), showed that the complete genome sequences of vB_KshKPC-M had no resemblance to the genome sequences recorded in Genebank. Phage vB_KshKPC-M is assigned to the *Siphoviridae*, order *Caudovirales*. It showed similarity to *Klebsiella* phages, *Klebsiella* phage 13 (with 86% similarity and 26.61% coverage) with accession code NC_049844.1, *Klebsiella* phage Sushi (with 83% similarity) and coverage 35.75%) with accession code NC_028774.1, *Klebsiella* phage vB_KpnD_PeteCarol (with 82% similarity and coverage 33.54%) with accession code OL539448.1 and *Klebsiella* phage PWKp14) with 82% similarity and coverage 53 / 5 33%) with accession code MZ634345.1 showed. All of these phages belonged to the *Siphoviridae* family.

**Fig. 14:**
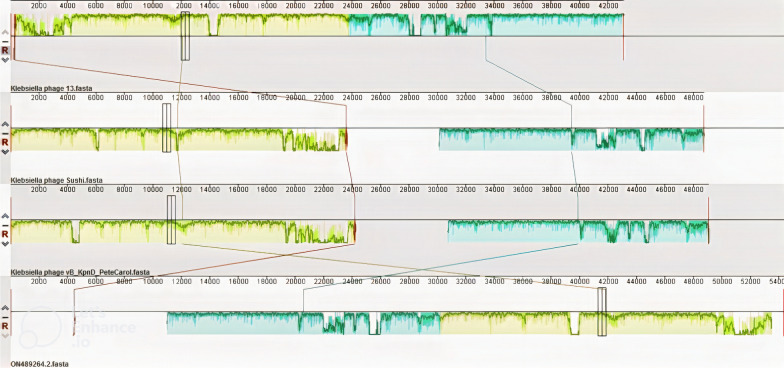
Alignment of vB_KshKPC-M phage genome with *Klebsiella* phages vB_KpnD_PeteCarol, Sushi, and phage 13. The colored blocks include similar regions between the phage genomes, and the height of the plates inside the blocks indicates the intensity of nucleotide similarity

**Fig. 15:**
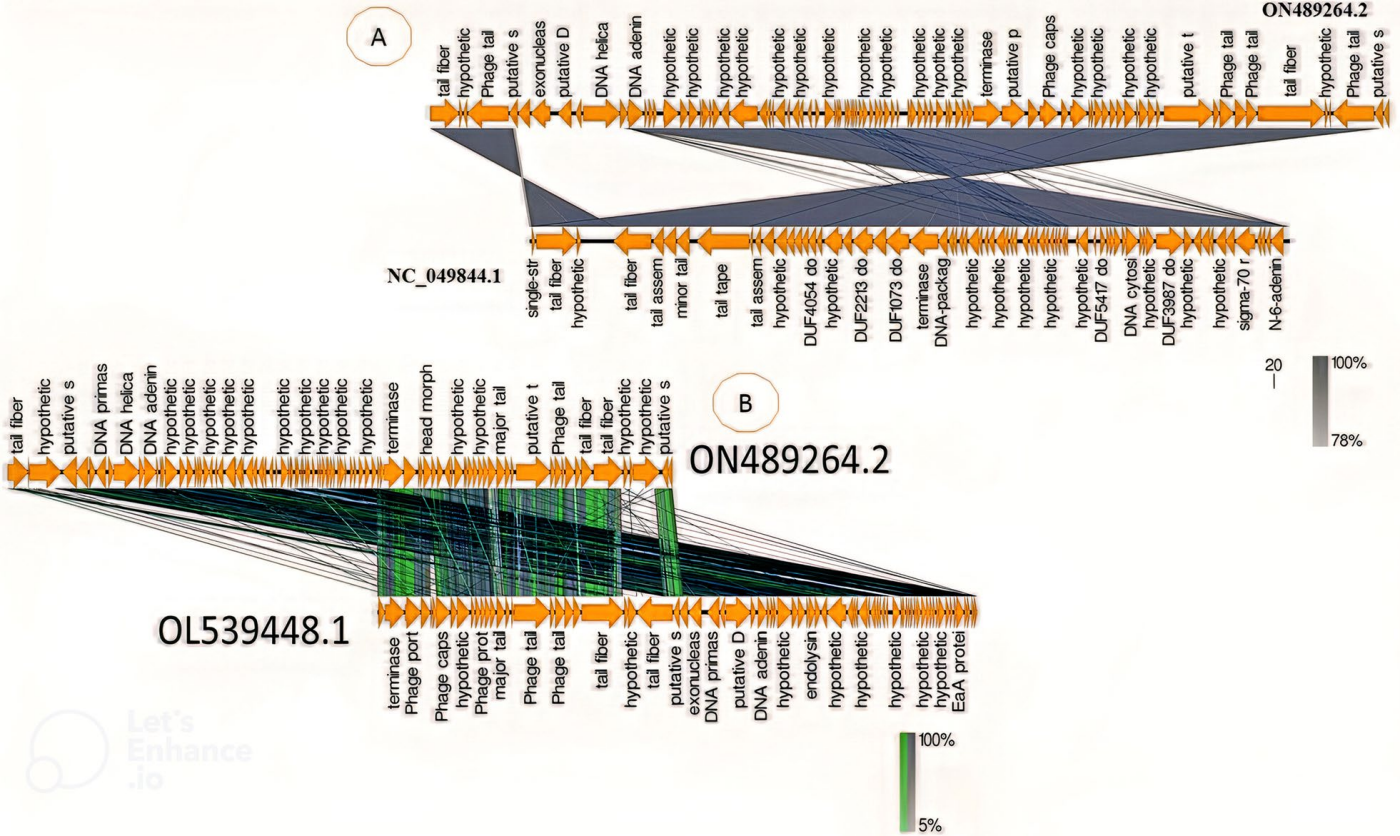
Comparison of vB_KshKPC-M phage genome with *Klebsiella* vB_KpnD_PeteCarol phage genome and phage 13 with EasyFig software. The arrows indicate the predicted ORFs in different colors according to their function in the genome. The profile of gene similarity between phages is shown as a gray color spectrum as a percentage. **A**: *Klebsiella* phage 13, **B**: *Klebsiella* phage vB_KpnD_PeteCarol

The complete genome sequence of phage vB_KshKPC-M was aligned with the whole genome of almost similar phages using Mauve software, and the results are shown in Fig. 14. Due to the low similarity of phage vB_KshKPC-M with other phages aligned using Mauve 2.4.0 software, some regions of the genome of phage vB_KshKPC-M resemble the *Klebsiella* phages shown in the figure, and these regions are marked as colored blocks in the figure. In addition, EasyFig software was used to compare the genome sequence of phage vB_KshKPC-M with *Klebsiella* phages vB_KpnD_PeteCarol, Sushi, and phage 13 (Fig. 15).

**Fig. 16:**
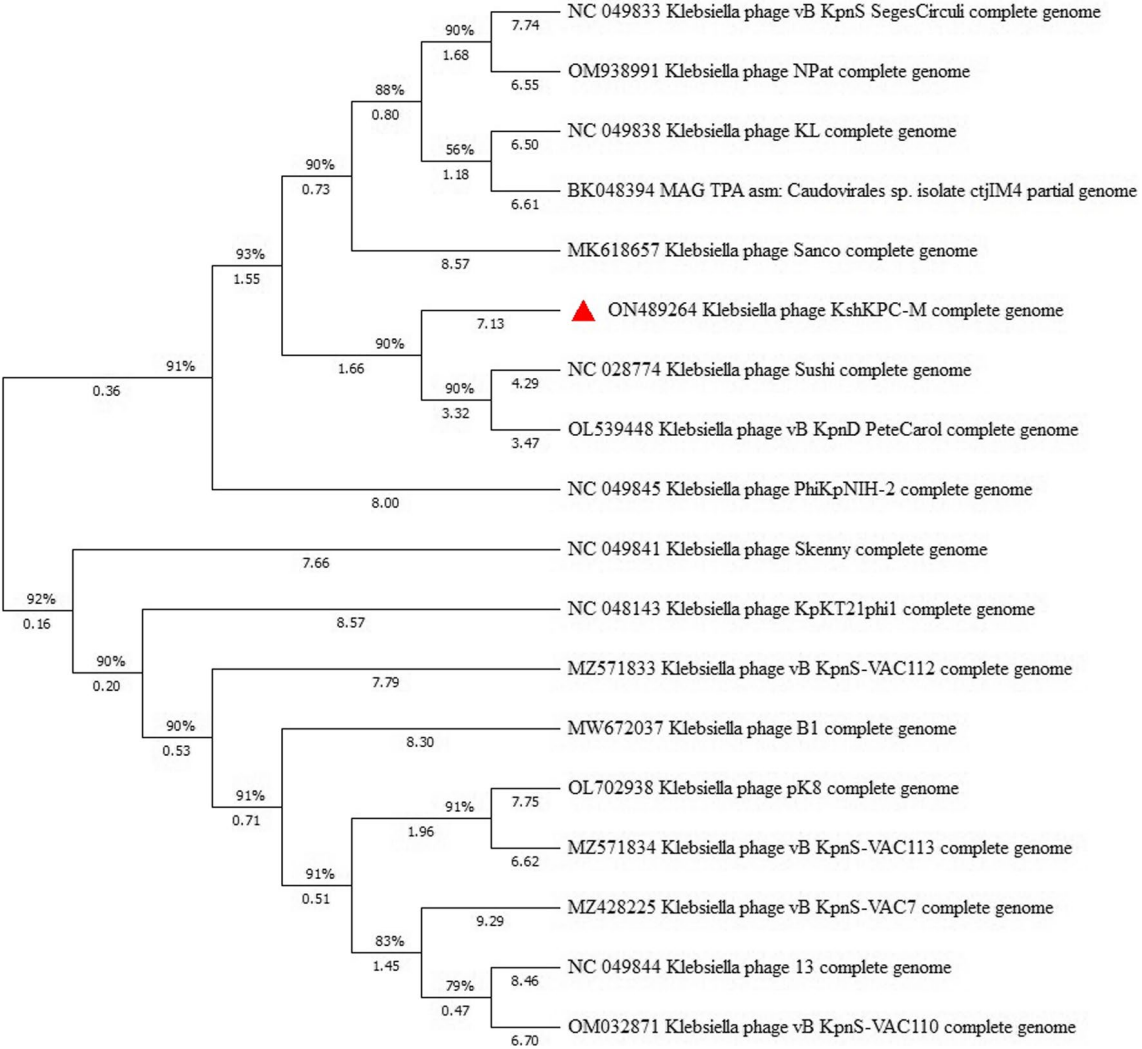
The phylogenetic tree of Phage vB_KshKPC-M. The phylogenetic tree was done with MEGA 11.0.13 software using the Maximum Likelihood method with the Bootstrap test method with the number of 1000 replicates using major capsid protein sequences and the Tamura 3-parameter model. The red symbol indicates our determined phage. Comparative genomic analysis revealed that phage vB_KshKPC-M has sequence similarity to the *Klebsiella* phages, phage 13 (NC_049844.1), phage Sushi (NC_028774.1), phage vB_KpnD_PeteCarol (OL539448.1) and phage PWKp14 (MZ634345.1)

In the phylogenetic tree analysis, the predicted proteins of the large subunit of the terminase and the major protein of the capsid were considered to verify the relatedness of phage vB_KshKPC-M with other similar phages in Genebank. The results of BLASTp analysis showed that the significant terminase subunit protein of phage vB_KshKPC M had 60% similarity to the major terminase subunit protein of *Klebsiella* phages vB_KpnD_PeteCarol, Sushi, and phage 13, and the major capsid protein was 61% similar to the main capsid protein of *Klebsiella* phage vB_KpnD_PeteCarol. It had 60 similarities. On this basis, the phylogenetic tree was constructed using MEGA 11 software (Fig. 16). Comparative genomic analysis revealed that phage vB_KshKPC-M has sequence similarity to the *Klebsiella* phages, phage 13 (NC_049844.1), phage Sushi (NC_028774.1), phage vB_KpnD_PeteCarol (OL539448.1) and phage PWKp14 (MZ634345.1). Phylogenetic analysis based on multiple alignments of the DNA polymerase and major capsid protein showed that vB_KshKPC-M is most closely related to the family *Siphoviridae*. The genome sequence of phage vB_KshKPC-M was deposited in the GenBank database under accession number ON489264.2.

## Discussion

Numerous studies have shown that phages are safe and effective in treating drug-resistant bacterial diseases [49–54]. Research and development of novel therapies based on phages and their encoding products could help treat infections caused by drug-resistant bacteria that are on the rise. [13]. In the present work, a phage that can lyse CRKP (VB KshKPC-M) was identified. Its biological properties, whole genome, and coding products were studied, and efficacy tests were performed.

Today, phage therapy is proposed as a suitable alternative to antibiotics in the treatment of many infectious diseases [55]. With the proliferation of CRKP strains in recent years, many researchers intend to use bacteriophage research for clinical purposes [17].

Following human clinical trials, phage lookup has evolved into in-vivo testing, which has sparked a political battle among regulatory organizations in Western nations. However, foreign sites in Eastern Europe and the former Soviet Union, for long years, had mechanically aged versions of their healthcare systems [1–3]. For instance, the Hirszfeld Institute of Immunology or Experimental Therapy in Poland and the Eliava Institute of Bacteriophages, Microbiology, and Virology in Georgia provide phage medicine items since they support moral purposes [1]. Phages are occasionally and only in exceptional circumstances used therapeutically inside the larger EU following Article 37 (Unproven Interventions into Clinical Practice) of the Helsinki Declaration [4–9].

Various reports are dedicated to clinical uses of phages in human samples and antibiotic crises in patients. After the patient visited the Eliava Institute for phage therapy, Dutch doctors reported successfully treating a renal transplant patient with a recurring urinary tract infection (UTI) caused by ESBL-producing *K. pneumoniae* using a combination of meropenem and phages [10]. Bacteriophage therapy approaches are used to treatment for orthopedic-related diseases [11]. Phagotherapy is applicable in treating burn patients in various clinical cases [12]. In clinical experience, a lytic phage was used against Carbapenem-resistant *Acinetobacter baumannii* lung infection in a Chinese man patient 88 years old with chronic obstructive pulmonary disease [13]. In another clinical experience of phage therapy, a 17 years old patient with cystic fibrosis was cured by lytic phage against *Achromobacter xylosoxidans* that cause lung infection [14]. There is a successful use of phage therapy in refractory MRSA chronic rhinosinusitis [15]. After multiple failed antibiotic treatments, chronic bacterial prostatitis was treated with phage therapy [16]. A report explains a combination of pre-adapted bacteriophage therapy and antibiotics for the treatment of fracture-related infection due to pan-drug-resistant *K. pneumoniae* [17]. Another clinical experience of phage therapy is the limb-threatening prosthetic knee *K. pneumoniae* infection by KpJH46Φ2 phage [18].

A case series report on phage therapy usage in infections [19]. We can assess more reports of clinical cases of phage therapy in various systematic reviews like the Aranaga et al. report [20] and Suh et al. report [21]. New reports mentioned bacteriophage therapy could potentially treat pediatric respiratory infections [22].

There are confident reports demonstrating that Phage therapy is safe and without phage-related adverse effects [23]. Phage therapy is safe and non-toxic in humans will be critical for their ultimate success [56]. But, there are also potential risks for phage therapy, such as the potential transfer of virulence or antibiotic resistance genes to infected bacteria, which may lead to such risks as the emergence of highly pathogenic strains. One of the main downsides of phage treatment is the increased safety concerns around using self-replicating living organisms in humans. For instance, as predicted, even while phages are colorful enough to cause antibiotic impediment, toxin-encoding genes can continue propagating to the target virus via transduction [24, 25]. Therefore, when assessing phages for medicinal applications, precise characterization is essential. Throughout this process, it is frequently screened for potentially dangerous genes. However, phage protection is no longer assured by the default of hazardous genes. For instance, lytic phages can multiply at the rate of their bacterial hosts. Though little research has been done on the dynamic side effects of that phenomenon, that is the main objective of phage treatment. Since phages, including a broad range but those present in a phage cocktail, are generally thought to conform to staying better suited because of phage therapy, safety is essential. What effects may phage medications have on an individual’s normal microbiota? Could the lessened risk of harm to the commensal microbiota make phages with a restricted host range, as indicated above, safer? If so, phage therapy must reject the appropriate phage readily accessible for treatment while remembering to identify the infecting bacteria with absolute precision. As much as the current antibiotic regimens are being litigated, that specific side effect could still be tolerated. Additionally, the safety of phage treatment for people has only been examined in a small number of clinical trials.

Phages that can lyse most bacterial strains, especially antibiotic-resistant ones, are the best candidates for alternative therapy in resistant isolates or can be used in combination therapy [17]. This report showed that the vB_KshKPC-M phage was able to lyse 44 isolates of CRKP isolates (97.7%).

The vB_KshKPC-M phage has the highest temperature stability at 37–45 °C. Its stability decreased with increasing temperature, so the bacteriophage became inactive at 80 °C. In addition, the phage exhibits high stability in various acidic and alkaline conditions, suggesting that its stability in body fluids and organelles may make it an excellent candidate for phage therapy. The maximum pH stability of the phage was in the physiological range (pH = 7). The pH stability of vB_KshKPC-M phage was utterly lost in a wide range of acidic and alkaline (pH 2 and 12).

In a study by Zhang et al., the temperature stability of phages was similar. It was found that the phage vB_KpnP_IME279 was most stable at 37 °C and had good stability at 45–65 °C, but at higher temperatures, the phage activity decreased significantly at 80 °C, and the phage became completely inactive. The Phage stability at neutral pH (pH = 7) is the same for both phages, but the acid range of the vB_KshKPC-M phage is lower (pH = 2), which is the strength of this phage over the vB_KpnP_IME279 phage. The same superiority of the vB_KshKPC-M phage is also present in the maximum alkaline range (pH = 12) [57].

In a later study in 2020, Shi et al. showed that the phage kpssk3 was stable at temperatures between 40 and 60 °C for an hour. However, the phage disappeared when incubated at 70 °C for 40 min. The range of temperature stability of the vB_KshKPC-M phage is better than that of the kpssk3 phage because the temperature range of the body is lower than the stability limit for the kpssk3 phage.

In addition, the stability of this kpssk3 phage at different pH values (2–10) showed that it was stable at pH values from 4 to 9, but its activity decreased dramatically at pH values of 10 and 3 and became completely inactive at pH 2. Compared with the present kpssk3 phage, the pH range stability of the vB_KshKPC-M phage also has a wide range, which is a significant advantage in using phages in medicine. [53].

Horváth et al. showed that phage vB_KpnS_Kp13 was stable at high temperatures (60 °C) and different pH (3–12). The results of this report were consistent with our report [58].

Feng et al. evaluated the performance of bacteriophage BUCT556A. The results showed that the lytic activity of phage was maintained at temperatures between 60 and 25 °C and lost its lytic activity at 70 °C. Its stability in the pH range was 4 to 13 [51]. The noteworthy point is that the temperature stability range of this BUCT556A phage is minimally lower than that of the vB_KshKPC-M phage, which can be considered an advantage for phage therapy. The conditions are the same in the pH range.

In the study by Gaoet al., the effect of bacteriophage vB_KpnP_IME337 against CRKP strains was determined. This study showed that this bacteriophage was stable at a temperature between 65 and 45 °C, but its lytic activity gradually decreased with increasing temperature. In addition, its performance range is with acids and bases [59]. Compared with this vB_KpnP_IME337 phage, the vB_KshKPC-M phage is superior in terms of temperature and pH stability.

In the present study, the vB_KshKPC M phage has a relatively short incubation time of about 20 min, and its phage burst size is about 260 phage particles per infected cell. These data confirm that the high production rate indicates the lytic nature of the isolated bacteriophages. Generally, a phage with a short incubation period and a high production rate are considered suitable for phage therapy. Therefore, the studied phage can be proposed as a right candidate for phage therapy.

Teng et al. reported the latency time by one-step growth chart for the phage Henu1 isolated against CRKP strains. The burst size was 30 min and 110 PFU per bacterial cell [60]. The vB_KshKPC-M phage burst size and latency time are shorter vs. Henu1phage that, which indicates vB_KshKPC-M phage can be a very suitable phage for phage therapy.

Another study estimating the latency of bacteriophage P509 was by Li et al. This phage was isolated against Carbapenem-resistant *K. pneumoniae,* and its burst size was 15 min and 85 PFU per bacterial cell [61]. Compared to this P509 phage, the vB_KshKPC-M phage has an advantage in terms of latency time and burst size for therapeutic selection.

In the study by D’Andrea et al., the ϕBO1E phage was isolated against carbapenem-resistant *K. pneumoniae* strains. The latency and burst size were 10 min and 300 PFU per bacterial cell, respectively [62]. Compared to this phage, the ϕBO1E phage has a higher ability than the vB_KshKPC-M phage. Comparing the above parameters with the above studies shows that the current vB_KshKPC-M phage can be used in phage therapy.

The different concentrations of calcium ions play an essential role in increasing the infectivity of bacteriophages. In addition, the calcium ion causes an increased accumulation of phage particles on the host cell's surface. The phage accumulation occurs by changing the structure of phage receptors on the cell surface. It increases the binding of phage and the transfer of its genome into the host cell. This ultimately leads to increased uptake of bacteriophage into the cell. Some divalent cations such as calcium (Ca^+2^), magnesium (Mg^+2^), and sugar compounds such as glucose play a role in phage binding and interaction with its specific receptors on the bacterial cell surface. Cofactors such as Ca^+2^ and Mg^+2^ cause the stability of initial and weak connections between virions with specific receptors during phage attraction to the host [63].

In this study, the rate of phage uptake to specific receptors of the host cell determined in the presence of 10 mM calcium chloride and magnesium chloride, which was significantly increased compared to the control sample (bacteriophage without divalent cation treatment). The results obtained in this study agree with the findings of Horváth [58]. They suggested that metal ions such as calcium and magnesium increase and stabilize bacteriophage absorption.

Phages have different ideal MOIs; the fewer phages needed to lyse the same amount of bacteria, the smaller the optimal MOI. Similar to the *Klebsiella* phage vB_KpnP_IME337, the ideal MOI of the vB_KshKPC-M phage MOI was 0.001, meanings that the phage unit produced the most progeny and had the best multiplication efficiency at this MOI. In the industrial production of phages, researchers often cultivate phages based on the corresponding MOI to produce a higher titer, which decreases production costs and boosts profitability [59].

Based on existing classifications and most studies performed, phages against carbapenemase-producing *K. pneumoniae* isolates are double-stranded DNA phages and belong to the four families of *Siphoviridae*, *Myoviridae*, *Podoviridae*, and *Autotrophiviridae* in the order *Caudovirales* [51, 64–68].

This phage classified as a member of the *Siphoviridae* family. The classification follows the most recent recommendations of the ICTV (International Committee on Taxonomy of Viruses, http://ictv.global/taxonomyRelease.s.asp). However, since the introduction of new phage families, phages can no longer be assigned to a specific family based on micrographs alone. The VB_KshKPC-M is comparable in head-to-tail proportions (the head diameter is approximately 35% of the tail length; Figs. 2E) to T1-like phage-like vB_EcoS_ACG-M12 [48].

VB_KshKPC-M can form transparent plaques with a halo of about 3 mm in diameter, and the plaques indicate that VB_KshKPC-M is a lytic phage [69]. As a lytic phage, VB_KshKPC-M has promising potential for clinical therapy and phage preparation. When bacterial growth on the plate reaches a plateau, phage proliferation usually stops or slows dramatically and does not increase the plaque area. However, the tails of some phages can still depolymerize bacterial exopolysaccharides, resulting in translucent areas of varying sizes on the outside of the phage plaque, referred to as halos [70]. A halo indicates that the phage has depolymerase activity, and depolymerase is a potential choice for treating antibiotic-resistant bacterial infections [71].

The phage VB_KshKPC-M possesses three typical lysis cassette genes encoding endolysin, holin, and spanin. The coordinated activity of endolysin, holin, and spanin leads to host lysis and progeny release, making VB_KshKPC-M an effective weapon with great lytic power against CRKP. The tail fiber protein sequence (ORF82) of VB_KshKPC-M showed similarity only to the *Klebsiella* phage vB_KpnP_IME279 [57], although the complete genome sequence of VB_KshKPC-M showed high similarity to 30 phages isolated from different parts of the world. This suggests an explanation for the host specificity of VB_KshKPC-M and confirms previous observations that highly homologous phages can exhibit significant differences in host-related genes.

## Conclusion

Since nosocomial infections, especially respiratory infections, are one of the most common causes in hospital intensive care units, this study aims to isolate lytic bacteriophage against CRKP strain Kp100, which is resistant to Colistin and has high potency for biofilm production. The isolated phage was able to lyse most of the antibiotic-resistant clinical isolates. Therefore, this phage can be used alone or as a phage mixture in future studies to control and inhibit respiratory infections caused by these bacteria, especially in treating respiratory infections caused by resistant strains in sick patients. The phage studied exhibited high stability to high temperatures and pH changes. They also had a fast absorption time and a high progeny production rate, which can introduce them as a suitable option for phage therapy. In this study, phage vB_KshKPC-M had high killing activity against CRKP strain, Kp100 in planktonic and biofilm forms.

Moreover, according to the results of the complete genome sequence, phage vB_KshKPC-M lacks genes encoding drug resistance, genes involved in bacterial virulence, and genes encoding the lysogenic cycle. Therefore, according to all the results obtained in this study, lytic bacteriophages can be considered powerful biological tools that should be proposed and used to treat respiratory infections, especially biofilms. The use of these bacteriophages in treating respiratory infections, especially biofilms, reduces the use of antibiotics and thus the cost of treatment and the length of hospital stay. The guiding significance of this study for clinical treatment of Carbapenem-resistant *Klebsiella pneumoniae* infection Due to the critical need for phage whole genome sequencing before any clinical use, the present study is the first study that has scientifically observed all the aspects mentioned in the article for the use of phage in treatment. This report is the first study that has been able to isolate, purify and prepare lytic phage against CRKP isolates for clinical use in Iran and the middle-east region. The mentioned phage has all the necessary properties for its use in the patient’s bed according to the laboratory criteria. Finally, There are little obstacles that stand in the way of implementation like as financial limitations for extend of our research and gaining more and more lytic phages with genome sequencing, and ethical restrictions in their use of them.

## Data Availability

The genome sequence of phage vB_KshKPC-M was deposited in the GenBank database under accession number ON489264.2.
